# Quadruplex Bioactive FAND for Treating Acute Liver Failure Induced by Acetaminophen or Hepatectomy

**DOI:** 10.1002/exp2.70204

**Published:** 2026-07-31

**Authors:** Meng Sun, Fang Fang, Juan Liu, Yueyun Fan, Sa Wang, Chuang Zhang, Weiyu Li, Zhengyang Quan, Dongxu Zhao, Min Hu, Jinfeng Zhang

**Affiliations:** ^1^ Key Laboratory of Molecular Medicine and Biotherapy School of Life Sciences Beijing Institute of Technology Beijing P. R. China; ^2^ Hepato‐Pancreato‐Biliary Center Key Laboratory of Digital Intelligence Hepatology (Ministry of Education) Beijing Tsinghua Changgung Hospital School of Clinical Medicine Tsinghua University Beijing P. R. China; ^3^ Department of Hepatobiliary Surgery Jinan University First Affiliated Hospital Guangzhou P. R. China

**Keywords:** anti‐inflammation, acute liver failure (ALF), full‐API nanodrug (FAND), fusion extracellular vesicles, hepatectomy

## Abstract

Acute liver failure (ALF), characterized by severe hepatocyte necrosis with a high mortality rate, remains a major global health challenge. However, there are currently no effective drug options for the clinical treatment of ALF. Herein, inspired by the new concept of a full‐API nanodrug (FAND), we have rationally developed a quadruplex bioactive FAND (termed FAND^HP@FuEVs^) composed entirely of active pharmaceutical ingredients (APIs). This FAND^HP@FuEVs^ is constructed from fusion extracellular vesicles (FuEVs), which hybridize M2 macrophage‐derived EVs (M2–EVs) with mesenchymal stem cell‐derived EVs (MSC‐EVs) and is subsequently engineered with two clinically therapeutic biomacromolecules: hepatocyte growth factor (HGF) and polyene phosphatidylcholine (PPC). Notably, FAND^HP@FuEVs^ efficiently targets the damaged liver, benefiting from the dual inherent inflammation‐tropism of the FuEVs. Moreover, FAND^HP@FuEVs^ harnesses quadruplex biological activities by leveraging four natural bioactive components—M2‐EVs, MSC‐EVs, HGF, and PPC—to deliver pleiotropic therapies, including antioxidant, anti‐inflammatory, pro‐regenerative, and macrophage repolarization effects. These therapies are effective in treating ALF induced by both acetaminophen and hepatectomy, demonstrating significant clinical relevance based on data from patients with liver disease. Overall, the utilization of naturally derived or clinically approved APIs to construct full‐bioactive nanodrugs creates opportunities for clinical translation as a safe, versatile, and multifaceted treatment for ALF.

## Graphical abstract

This work developed a bioactive quadruplex FAND^HP@FuEVs^ with intrinsic inflammation‐targeting properties of M2‐EVs and MSC‐EVs, harnessing the synergistic biological activities by leveraging four natural active components—M2‐EVs, MSC‐EVs, HGF, and PPC—to deliver pleiotropic therapies, including antioxidant, anti‐inflammatory, pro‐regenerative, and M1–M2 repolarization effects, for the treatment of APAP‐ and hepatectomy‐ induced ALF both in vivo and in vitro.

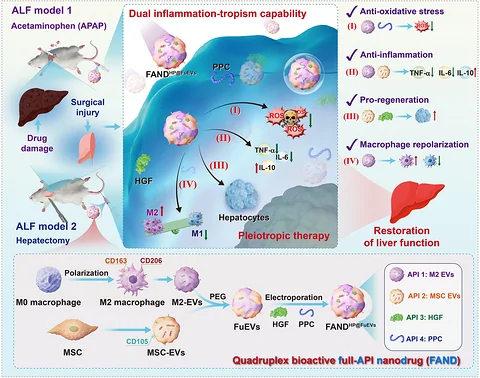

## 1 Introduction

Acute liver failure (ALF) is accompanied by overwhelming hepatocyte necrosis, acute liver dysfunction, and life‐threatening complications, resulting in a critical global health challenge with a high mortality rate [1, 2, 3, 4]. The lethal condition of ALF is typically triggered by various factors, such as drug‐induced damage, surgical resection, and autoimmune disorders [5, 6, 7]. Although artificial liver support systems and orthotopic liver transplantation are considered effective therapy options for the treatment of ALF in the clinic, both of them face severe obstacles, such as donor scarcity, high surgical costs, and the need for lifelong immunosuppression [8, 9]. More critically, there are currently no effective drug‐based interventions for the clinical treatment of ALF. Thus, continuous efforts to develop a safe, efficient, and widely applicable restorative treatment for ALF remain highly essential.

Recently, advanced nanodrugs have shown promising potential in both fundamental research and clinical applications for ALF therapy [10, 11, 12, 13]. However, traditional nanodrugs are often hampered by three major limitations: (1) Low active pharmaceutical ingredient (API) loading capacity (typically below 10 wt.%) due to a high proportion of non‐therapeutic components (such as structural lipids or excipients); (2) Biosafety concerns associated with inert carriers (including mesoporous silica) and complex surface modifiers that exhibit poor biodegradability and pose challenges for long‐term clearance; and (3) Suboptimal therapeutic efficacy, stemming from reliance on monotherapy and insufficient targeting, particularly in systems that depend solely on the enhanced permeability and retention (EPR) effect, which is highly heterogeneous across patients and disease states [14, 15, 16]. Recently, the concept of a full‐API nanodrug (FAND), with a 100% API composition, has been newly proposed, offering leapfrogging opportunities in nanoformulations to address these limitations [17]. However, currently available FANDs are commonly composed of FDA‐approved small molecules and human essential metal ions, which exhibit low bioactivity and limited biomimetic functions [18, 19, 20]. With emerging supremacy, extracellular vesicles (EVs) naturally secreted by all types of cells present a promising alternative, as they offer inherent bioactive properties, excellent biocompatibility, low immunogenicity, and specific biofunctions originating from their parent cells [21, 22, 23, 24, 25, 26], making them powerful API building blocks for the construction of superior bioactive FANDs.

**SCHEME 1:**
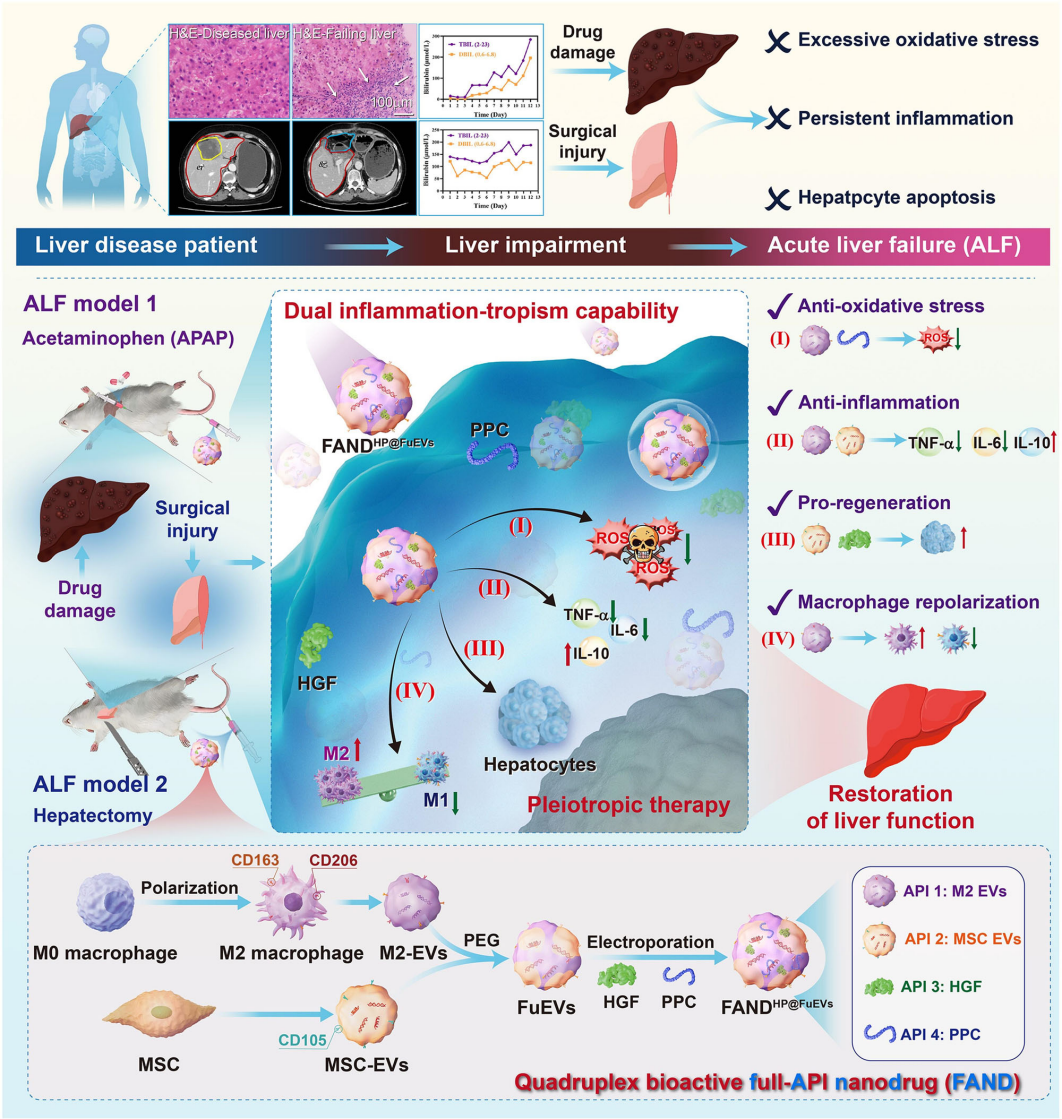
Schematic illustration of the design and pleiotropic biofunctions of quadruplex bioactive FAND (FAND^HP@FuEVs^) for the treatment of both acetaminophen (APAP)‐ and hepatectomy‐induced acute liver failure (ALF).

In this work, we have rationally designed and fabricated a quadruplex bioactive FAND (termed FAND^HP@FuEVs^), which consists of fusion EVs (FuEVs) engineered with two clinically therapeutic biomacromolecules, hepatocyte growth factor (HGF) and polyene phosphatidylcholine (PPC), for the effective treatment of both acetaminophen (APAP)‐ and hepatectomy‐induced ALF (Scheme 1). Specifically, FuEVs are engineered by fusing M2 macrophage‐derived EVs (M2‐EVs) with mesenchymal stem cell‐derived EVs (MSC‐EVs). Both EV subtypes carry chemotactic factors and display functional chemotactic receptors derived from their parent cells, enabling them to participate in inflammatory cue–directed trafficking and immune cell recruitment. This inherent inflammation‐tropic capacity is retained in the fused FAND^HP@FuEVs^, endowing the hybrid system with enhanced targeting and accumulation at sites of liver injury. Notably, the as‐prepared FAND^HP@FuEVs^ integrate four bioactive components that act through distinct yet complementary mechanisms to orchestrate liver repair: (1) antioxidant activity and M1‐to‐M2 macrophage repolarization mediated by M2‐EVs [27, 28, 29]; (2) anti‐inflammatory action and promotion of hepatocyte regeneration conferred by MSC‐EVs [30, 31, 32, 33]; (3) stimulation of hepatocyte growth and tissue repair driven by HGF, a pleiotropic cytokine that has demonstrated therapeutic efficacy in clinical trials for liver cirrhosis and ischemia‐reperfusion injury [34, 35, 36]; and (4) potent antioxidant and hepatoprotective effects provided by PPC, an FDA‐approved phospholipid formulation used to restore hepatocellular integrity [37, 38]. Collectively, these bioactivities confer a pleiotropic therapeutic effect on ALF both in vitro and in vivo. Importantly, based on clinical data indicating that liver failure often arises from drug toxicity or surgical injury in patients with liver disease, the FAND^HP@FuEVs^ have been validated in both APAP‐ and hepatectomy‐induced ALF animal models, demonstrating great clinical significance. In short, the current quadruplex bioactive FAND^HP@FuEVs^ delicately integrate four natural bioactive components—M2‐EVs, MSC‐EVs, HGF, and PPC, providing a safe, efficient, universal, and pleiotropic paradigm for the treatment of ALF and other inflammatory diseases.

## 2 Result and Discussion

### 2.1 Identification of Drug Damage or Surgical Injury in ALF Patients

**FIGURE 1:**
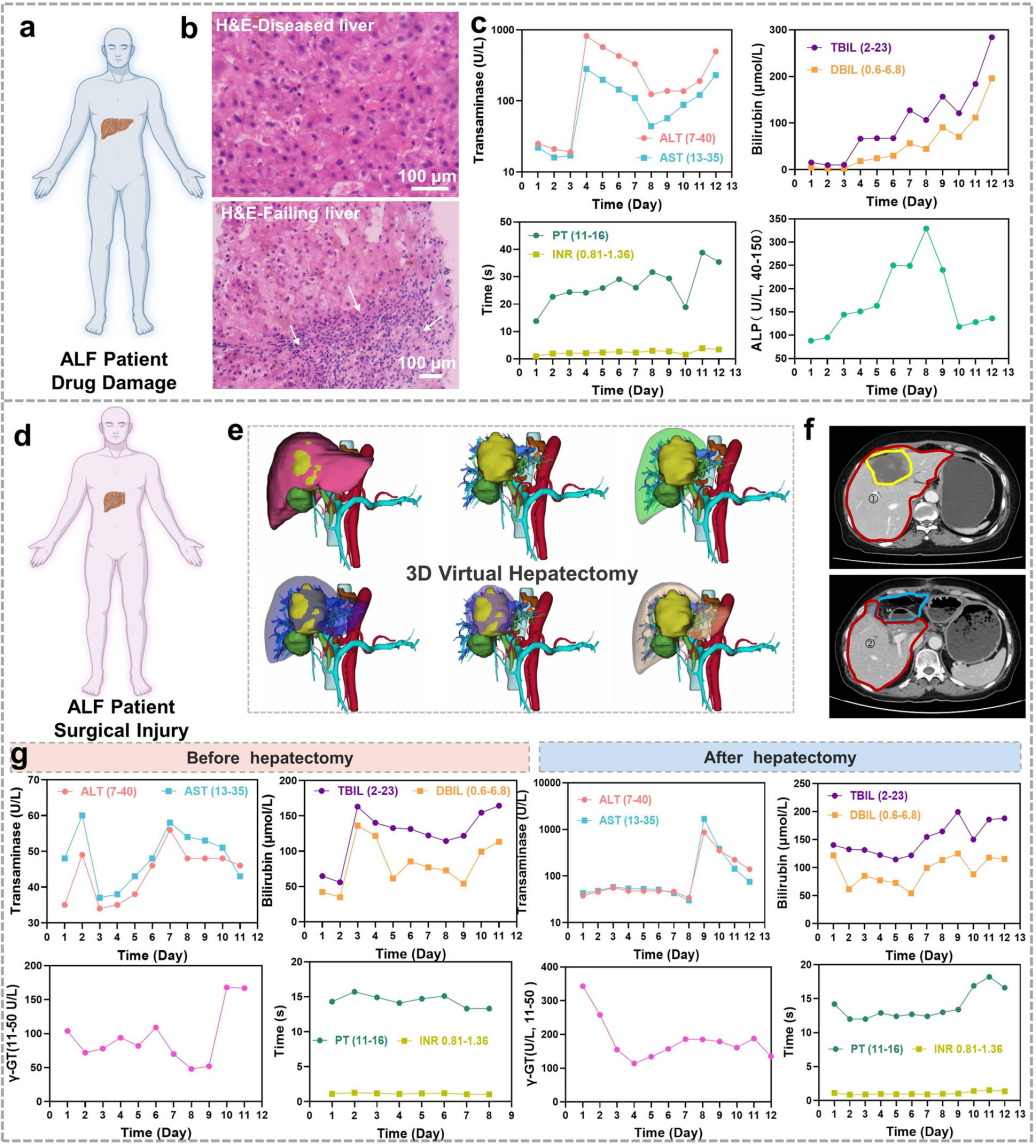
Identification of drug‑induced or surgical resection‑induced injury in ALF patients. (a) A representative ALF patient with drug‑induced injury; (b) H&E‑stained liver section from a patient with drug‑induced ALF. Scale bar = 100 µm; (c) changes in serum ALT, AST, TBIL, DBIL, PT, INR, and ALP levels after drug‑induced injury; (d) A representative ALF patient with surgical injury; (e) 3D virtual hepatectomy shows whole liver, tumor, and liver duct structure (red: artery, light green: portal vein, dark green: dilated bile duct, blue: hepatic vein); (f) postoperative CT image of a patient after hepatectomy (red: preserved liver, yellow: tumor, blue: resected liver); and (g) paired comparison of serum ALT, AST, TBIL, DBIL, γ‑GT, PT, and INR levels before and after hepatectomy.

To investigate ALF‐related damage in patients and establish a foundation for developing two ALF mouse models, we collected clinical data from patients suffering from drug‐induced injury or undergoing hepatectomy and evaluated several liver function indicators closely related to ALF. Firstly, histological examination revealed notable inflammatory cell infiltration in the liver sections of drug‐induced ALF patients (Figure 1a,b). After drug‐induced injury, alanine aminotransferase (ALT), aspartate aminotransferase (AST), total bilirubin (TBIL), direct bilirubin (DBIL), prothrombin time (PT), international normalized ratio (INR), and alkaline phosphatase (ALP) levels were significantly elevated beyond the normal range, indicating a potential risk of liver failure (Figure 1c). Additionally, as illustrated in Figure 1d–f, 3D virtual hepatectomy was used to assess surgical strategies for patients with liver cancer, with CT images delineating the tumor and the site of liver resection. Furthermore, Figure 1g compared the levels of ALT, AST, γ‐glutamyl transpeptidase (γ‐GT), TBIL, DBIL, PT, and INR before and after surgical resection. The significant increase in these indices indicated marked hepatic damage and consequent dysfunction. These clinical findings demonstrate that both drug damage and surgical injury can lead to liver failure, providing a solid basis for the development of APAP‐induced and hepatectomy‐induced ALF animal models. Notably, APAP, which is a common antipyretic and analgesic [39, 40, 41], often leads to ALF upon overdose.

### 2.2 Preparation and Characterization of M2‐EVs, MSC‐EVs, FuEVs, and FAND^HP@FuEVs^

**FIGURE 2:**
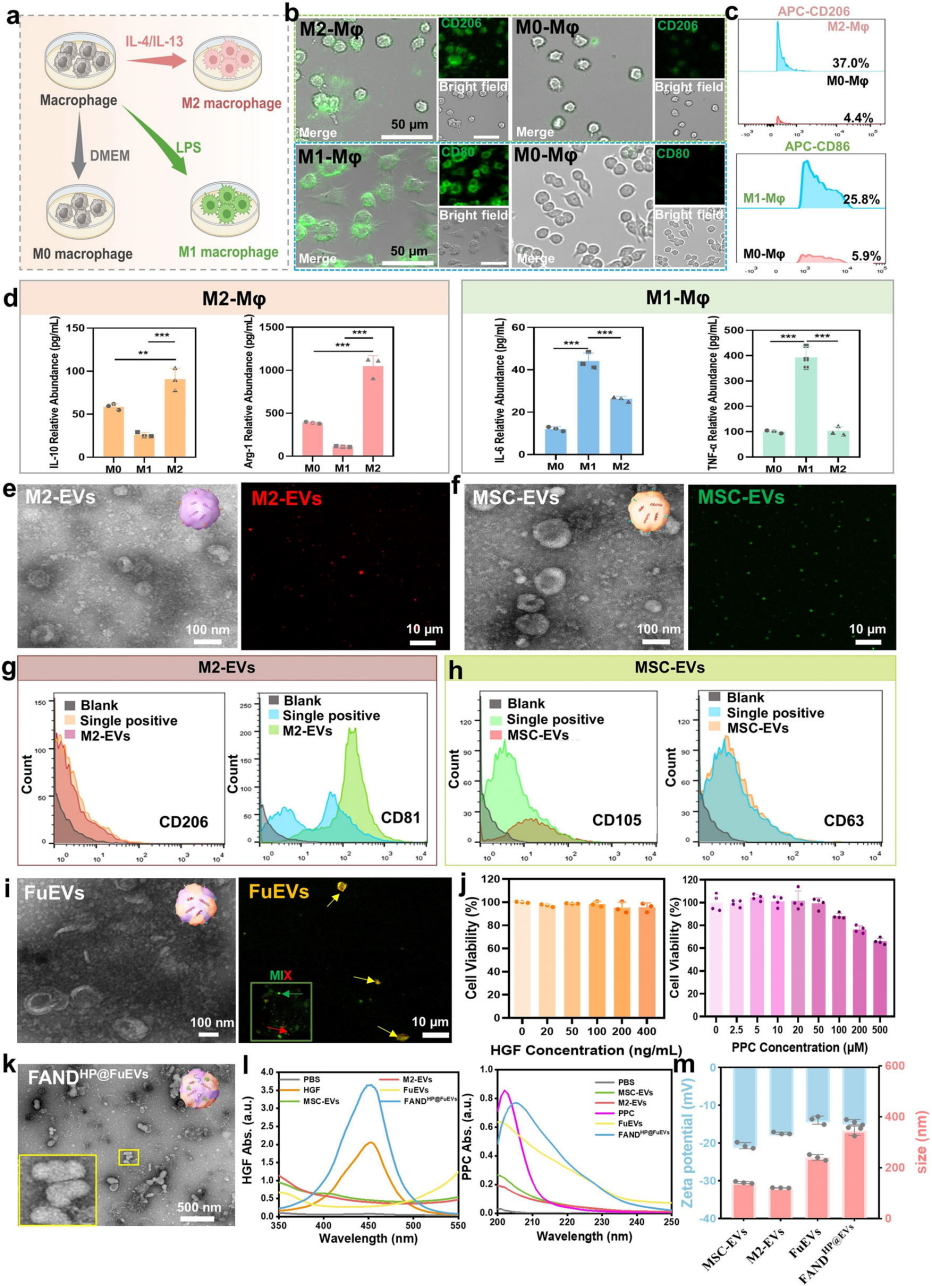
Preparation and characterization of M2‐EVs, MSC‐EVs, FuEVs, and FAND^HP@FuEVs^. (a) Schematic diagram of macrophage polarization; (b) CLSM images of M2 (FITC‐anti‐mouse CD206) and M1 (FITC‐anti‐mouse CD80) macrophage polarization; (c) flow cytometry analysis of CD206 and CD86 expressed on M2 and M1 macrophages; (d) changes in the levels of the M2 macrophage markers IL‐10 and Arg‐1, and the M1 macrophage markers IL‐6 and TNF‐α. *p < 0.05; **p < 0.01; ***p < 0.001; (e) TEM images and confocal imaging of M2‐EVs; (f) TEM images and confocal imaging of MSC‐EVs; (g) flow cytometry analysis of CD206 and CD81 expressed on M2‐EVs; (h) CD105 and CD63 expressed on MSC‐EVs (single‑positive controls represent marker proteins labeled with the same fluorophore as used in the experimental groups); (i) TEM images and confocal imaging of FuEVs (inset: vesicle mixture group); (j) cytotoxicity under varying concentrations of HGF (0, 20, 50, 100, 200, and 400 ng mL^−1^) and PPC (0, 2.5, 5, 10, 20, 50, 100, 200, and 400 µM). (k) TEM images of FAND^HP@FuEVs^; (l) UV absorption curve of HGF and PPC; and (m) size and zeta potential of M2‐EVs, MSC‐EVs, FuEVs, and FAND^HP@FuEVs^ (*n* = 3).

Of the three types of macrophages, M1 macrophages possess pro‐inflammatory properties, whereas M2 macrophages play a pivotal role in inhibiting oxidative stress and modulating the inflammatory microenvironment [42]. Similar to parental M2 macrophages, M2‐EVs inherit antioxidant and inflammation‐targeting effects, which are promising for use as an alternative to live cells in developing therapies with equivalent or superior efficacy. Additionally, MSC‐EVs not only have inflammation‐targeting and anti‐inflammatory effects but are especially renowned for their ability to promote cell regeneration in disease treatment [43, 44, 45]. Therefore, to isolate the M2‐EVs, mouse M0‐type macrophages were first polarized into M2‐type macrophages under the induction of IL‐4 (100 ng mL^−1^) and IL‐13 (100 ng mL^−1^) for 24 h [28, 46] (Figure 2a). Meanwhile, after treatment with LPS (1 µg mL^−1^) for approximately 24 h [47], M1 macrophages were obtained for subsequent experiments. As shown in Figures 2b and S2a, confocal laser scanning microscopy (CLSM) was used to observe the marker proteins CD206 (M2) and CD80/CD86 (M1) in the two cell types. Flow cytometry was then performed to determine the polarization ratios of the two cell types, which were found to be 37 and 25.8%, respectively (Figure 2c). Subsequently, an ELISA was performed to quantify the levels of the pro‐inflammatory M1 macrophage cytokines interleukin‐6 (IL‐6) and tumor necrosis factor‐alpha (TNF‐α) and the anti‐inflammatory M2 macrophage cytokine interleukin‐10 (IL‐10) in culture supernatants, as well as the M2 marker protein arginase 1 (Arg‐1), before and after polarization. The significant results demonstrated successful polarization of both M2 and M1 macrophages (Figures 2d and S1). After that, differential ultracentrifugation [48, 49] was employed to extract the M2‐EVs and MSC‐EVs. Transmission electron microscopy (TEM) images revealed the classic morphology of both types of EVs, while CLSM imaging using markers specific to each vesicle type confirmed their successful extraction (Figure 2e, f). Furthermore, flow cytometry was conducted to analyze the EV marker proteins CD81 and CD63, as well as the M2‐EVs marker CD206 and the MSC‐EVs marker CD105. Both types of EVs displayed a rich presence of these markers, confirming the successful extraction of M2‐EVs and MSC‐EVs (Figures 2 g, h, and S2).

Compared with single EVs, fusion EVs can enhance the inflammatory targeting capability and synergistically boost the therapeutic efficacy in treating ALF [50]. Meanwhile, the MW of PEG is a critical determinant of its fusion efficiency, which needs to be a moderate MW (1500–6000 Da) for membrane fusion, as it adequately dehydrates the membrane without excessive steric hindrance [51, 52]. Therefore, FuEVs were prepared by PEG 4000‐induced fusion of M2‐EVs and MSC‐EVs. Subsequently, two clinically therapeutic biomacromolecules, including HGF and phosphatidylcholine (PPC), were loaded via electroporation, resulting in the fabrication of a FAND with quadruple bioactivity, designated FAND^HP@FuEVs^. TEM characterized the morphology of FuEVs, and both CLSM imaging of FuEVs and the vesicle mixture group (MIX) and fluorescence resonance energy transfer (FRET) results of the three EVs confirmed the successful fusion of M2‐EVs and MSC‐EVs (Figures 2i and S3). In addition, the optimal concentrations of HGF (200 ng/mL) and PPC (100 µM) were determined using the MTT assay, ensuring that cell viability exceeded 80% (Figure 2j). Then, the TEM morphology of FAND^HP@FuEVs^ was observed in Figure 2k. Additionally, the successful loading of HGF and PPC was confirmed by ELISA and UV‐Vis spectroscopic analysis (Figure 2l). Quantitative assessment of drug encapsulation efficiency and drug loading efficiency was provided in Table S1 and Figure S4. Although multi‐concentration PPC absorption curves (Figure S4d) demonstrated a consistent red shift correlated with increasing drug concentration, PPC loading capacity and encapsulation efficiency were calculated using a standard calibration curve derived from UV spectra of pure PPC, which exclusively tracks PPC‐specific wavelengths unaffected by EVs or by other interference. Furthermore, in Figure 2m, nanoparticle tracking analysis (NTA) showed that the average diameters of the M2‐EVs, MSC‐EVs, FuEVs, and FAND^HP@FuEVs^ were 121.5 nm, 142.3 nm, 240.9 nm and 346.1 nm, respectively, while the surface zeta potential of the above four types of EVs was approximately −17.4 mV, −20.7 mV, −13.9 mV and −14.5 mV. Compared with single EVs, the diameters of the FuEVs and FAND^HP@FuEVs^ increased. Besides, the FuEVs and FAND^HP@FuEVs^ displayed outstanding stability in PBS (Figure S5). These results demonstrate that we successfully extracted and fused M2‐EVs with MSC‐EVs to fabricate FAND^HP@FuEVs^ with a suitable diameter and outstanding stability.

### 2.3 Comparison of miRNAs in Fusion Vesicles (FuEVs) and Single Vesicles (M2‐EVs and MSC‐EVs) by Sequencing Analysis

**FIGURE 3:**
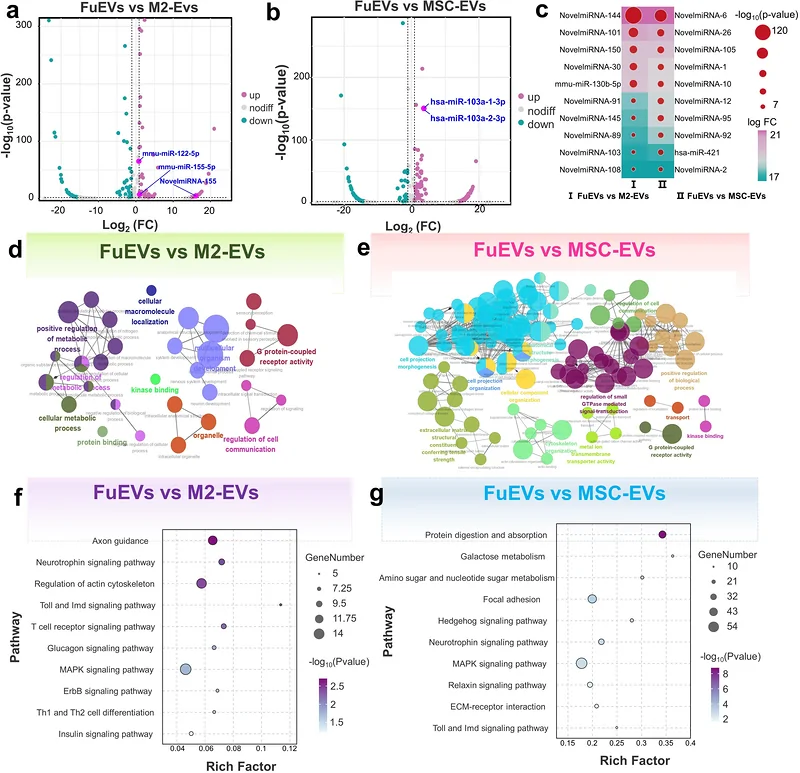
Analysis and comparison of miRNAs in fusion vesicles and single vesicles (M2‐EVs and MSC‐EVs). (a) Volcano plot of differentially expressed miRNAs between FuEVs and M2‐EVs; (b) volcano plot of differentially expressed miRNAs between FuEVs and MSC‐EVs; (c) bubble plots of ten up‐regulated miRNA target genes in the two comparison groups; (d) GO enrichment network of differentially expressed miRNA target genes between FuEVs and M2‐EVs; (e) GO enrichment network of differentially expressed miRNA target genes between FuEVs and MSC‐EVs; (f) KEGG pathway enrichment analysis of differentially expressed miRNA target genes between FuEVs and M2‐EVs; and (g) KEGG pathway enrichment analysis of differentially expressed miRNA target genes between FuEVs and MSC‐EVs.

As miRNA sequencing becomes increasingly instrumental in deciphering the therapeutic mechanisms of nanodrugs [53, 54, 55], we conducted a comparative analysis of the FuEVs and single EVs to investigate the differences between the prepared FuEVs and single EVs (M2‐EVs and MSC‐EVs), as well as the underlying regulatory pathways involved in treating ALF. First, we analyzed the high‐quality sequencing data (Figures S6 and S7) from three types of vesicles. Subsequently, the volcano plots showed differences in miRNA expression between the two compared groups. In Figure 3a, compared with M2‐EVs, the expression of miR‐155 and miR‐122, which are involved in liver regeneration and promote hepatocyte proliferation, was up‐regulated in FuEVs. Additionally, compared with MSC‐EVs, the up‐regulation of miR‐103 was most pronounced in FuEVs, modulating the hepatic microenvironment and alleviating fatty liver (Figure 3b), thereby laying the foundation for promoting liver regeneration and repair. Subsequently, we applied screening criteria (fold change > 1, adjusted *p* < 0.05) to identify predicted target genes of the up‐regulated miRNA in the two compared groups. Among them, the number of miRNAs up‐regulated target genes in the FuEVs and M2‐EVs comparison was 592, while that for FuEVs versus MSC‐EVs was 2579. Based on these results, we summarized the top ten target genes with the highest upregulation fold changes in Figure 3c to further analyze the genes. Gene Ontology (GO) enrichment analysis revealed molecular function, cell composition, and biological processes associated with the up‑regulated target genes in FuEVs compared with single vesicles. These were significantly enriched in terms related to positive regulation of biological processes, G protein‑coupled receptor signaling, cell communication, and cellular macromolecule localization (Figure 3d, e). In addition, a scatter plot illustrated the most significantly enriched Kyoto Encyclopedia of Genes and Genomes (KEGG) pathways. As shown in Figure 3f, g, the target genes involved in the pathways directly or indirectly affect hepatocyte repair. For example, the MAPK signaling pathway regulates cell proliferation, axon guidance controls hepatic arteriolar regeneration, and the protein digestion and absorption pathway is involved in systemic nutrient supply. These analyses confirm the rationale and efficacy of FuEVs for hepatocyte proliferation, metabolic regulation, drug delivery, and ALF treatment.

### 2.4 Uptake and Restoration Effects of FAND^HP@FuEVs^ on Damaged Hepatocytes

**FIGURE 4:**
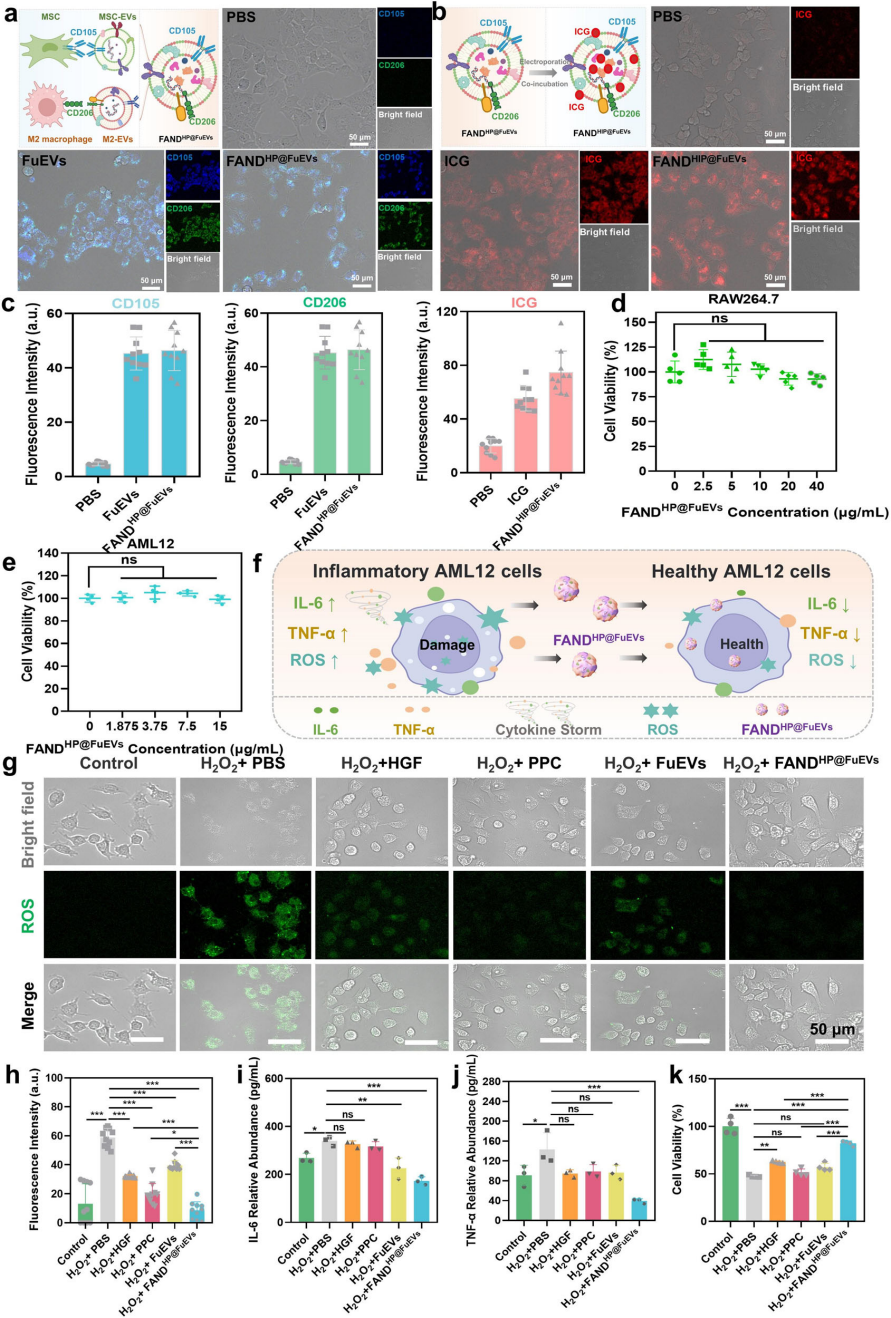
Uptake and restoration effects of FAND^HP@FuEVs^ on damaged hepatocytes. (a) Confocal imaging of FuEVs and FAND^HP@FuEVs^ uptake by AML12 cells; (b) confocal imaging of ICG and ICG‐loaded FAND^HIP@FuEVs^ uptake by AML12 cells; (c) fluorescence intensity of CD206, CD105, and ICG in AML12 cells; (d) cytotoxicity analysis of FAND^HIP@FuEVs^ on AML12; and (e) RAW264.7 cells; (f) schematic illustrating changes in IL‐6, TNF‐α, and ROS levels in inflammatory hepatocytes before and after FAND^HIP@FuEVs^ treatment; (g) representative images depicting ROS levels (green) stained with DCFH–DA in various treatment groups (Control, H_2_O_2_, H_2_O_2_ + HGF, H_2_O_2_ + PPC, H_2_O_2_ + FuEVs, and H_2_O_2_ + FAND^HP@FuEVs^) in AML12 cells. Scale bar = 50 µm; (h) mean fluorescence intensity of ROS in each treatment group; (i) Levels of IL‐6; and (j) TNF‐α in the supernatant of each treatment group; (k) cell viability across different treatment groups. *p < 0.05; **p < 0.01; ***p < 0.001.

The bioactive FAND^HP@FuEVs^ are expected to be taken up by damaged hepatocytes to release biomacromolecules and exert effective therapeutic effects. Therefore, the marker proteins CD206 and CD105 from M2 macrophages and MSCs were used to observe the uptake of FAND^HP@FuEVs^ by Alpha Mouse Liver 12 (AML12) cells. In Figure 4a, CLSM images demonstrated that AML12 cells displayed fluorescence from both types of vesicle marker proteins, indicating successful uptake of FuEVs and FAND^HP@FuEVs^. Meanwhile, a bioactive nanodrug loaded with ICG (FAND^HIP@FuEVs^) was prepared by electroporation and co‐incubation (Figure S8), and the CLSM images and fluorescence signal intensity of ICG in AML12 cells were observed to further confirm the successful uptake of FAND^HP@FuEVs^ by AML12 (Figure 4b, c). Next, we assessed the cytotoxicity of FAND^HP@FuEVs^ on two healthy cell types, AML12 and RAW264.7 cells. As illustrated in Figure 4d, e, the prepared FAND^HP@FuEVs^ exhibited negligible cytotoxic effects on both cell types across various concentrations and even stimulated cell growth (Figure S9), indicating excellent biosafety at the cellular level.

Excessive oxidative stress is a hallmark of ALF, characterized by significantly elevated reactive oxygen species (ROS) levels that lead to hepatocellular damage [56, 57]. To simulate an oxidative stress microenvironment, AML12 cells were treated with hydrogen peroxide (H_2_O_2_) to induce ROS production [58, 59] (Figure S10). This setup facilitated the evaluation of the antioxidant effects of the various components of FAND^HP@FuEVs^ at the cellular level. Moreover, Figure S11 further verified that vesicles (particularly FuEVs) are preferentially internalized by inflamed hepatocytes over healthy cells. Notably, FAND^HP@FuEVs^ exhibit significantly greater cellular uptake than individual MSC‐EVs or M2‐EVs, confirming that fusion engineering enhances intrinsic targeting capabilities. As illustrated in Figure 4f, the schematic diagram summarizes the changes in inflammatory cytokine levels and ROS content in inflammatory hepatocytes before and after FAND^HP@FuEVs^ treatment. CLSM images, quantitative analysis of the ROS fluorescence, and flow cytometric results demonstrated that the different components exhibited antioxidant capacity, with FAND^HP@FuEVs^ showing particularly remarkable efficacy in ROS clearance, attributed to the synergistic effects of the multiple components (Figures 4 g, h and S12). Moreover, Figure 4i, j showed the changes in the levels of pro‐inflammatory cytokines IL‐6 and TNF‐α following treatment with FAND^HP@FuEVs^ and each component. The results indicated downregulation of IL‐6 and TNF‐α expression levels, demonstrating that FAND^HP@FuEVs^ exerted a potent anti‐inflammatory effect at the cellular level. Furthermore, the CCK‐8 assay validated the effective repair of inflammatory hepatocytes by FAND^HP@FuEVs^ (Figure 4k). These results further demonstrate that the bioactive FAND^HP@FuEVs^, through the synergistic effects of their components (HGF, PPC, and FuEVs), exhibit potent antioxidant and anti‐inflammatory repair capacity at the cellular level.

### 2.5 Repolarization and Regenerative Effects of FAND^HP@FuEVs^ on Macrophages and Damaged Hepatocytes

**FIGURE 5:**
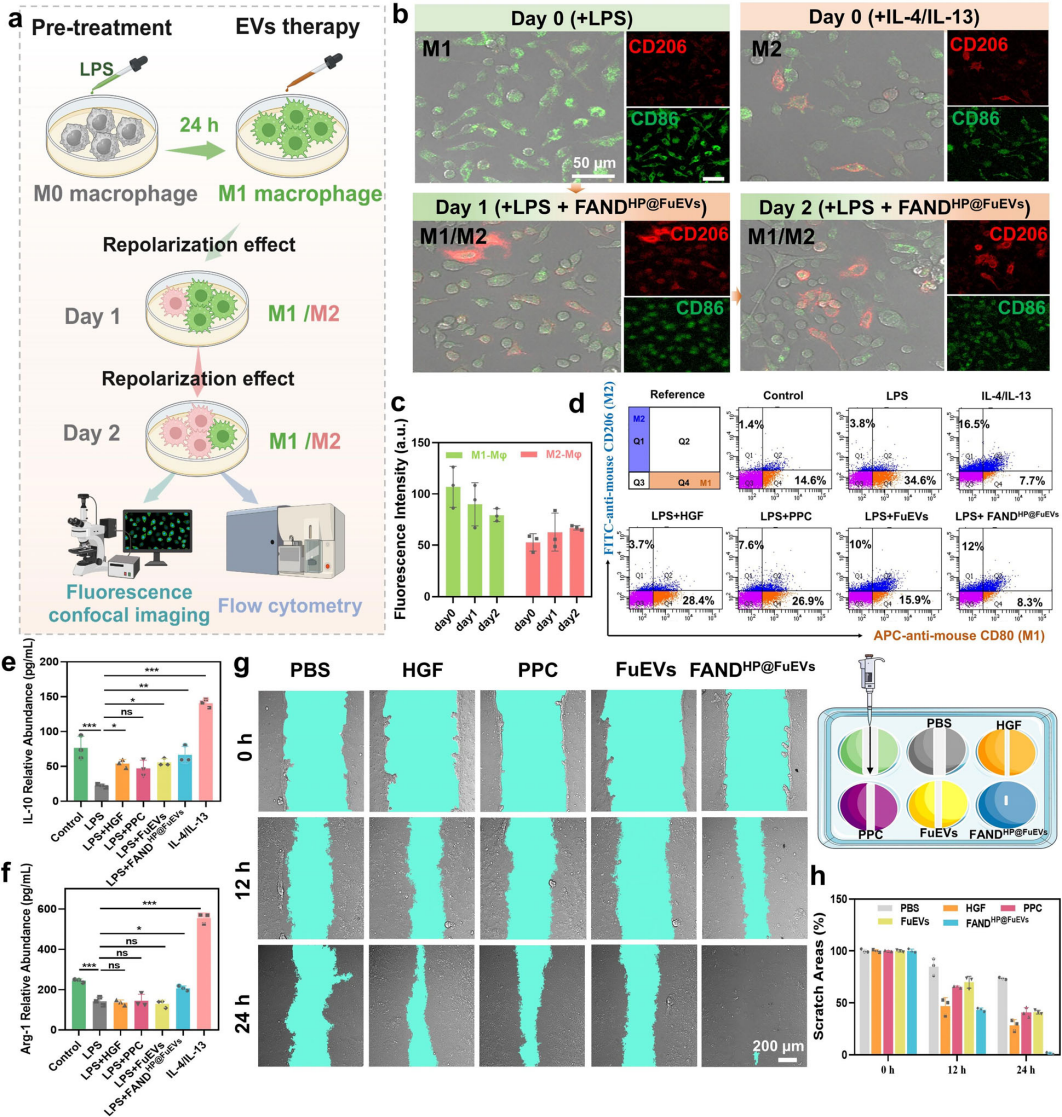
Repolarization and regenerative effects of FAND^HP@FuEVs^ on macrophages and damaged hepatocytes. (a) Schematic of the experimental workflow for validating the repolarization effect of FAND^HP@FuEVs^; (b) confocal imaging of M1 and M2 macrophage marker proteins during FAND^HP@FuEVs^‐induced repolarization. Scale bar = 50 µm; (c) changes in fluorescence intensity of M1 and M2 macrophages during FAND^HP@FuEVs^‐induced repolarization; (d) flow cytometric analysis of the repolarization effect of FAND^HP@FuEVs^; (e) changes in IL‐10; and (f) Arg‐1 levels upon FAND^HP@FuEVs^ treatment; (g) changes in the scratch area of healthy cells treated with FAND^HP@FuEVs^ and individual components; and (h) statistical analysis of scratch area changes for FAND^HP@FuEVs^ and component treatment groups. *p < 0.05; **p < 0.01; ***p < 0.001.

Based on the pro‐inflammatory role of M1 macrophages and the anti‐inflammatory effects of M2 macrophages in the damaged hepatic microenvironment, promoting the repolarization of M1 to M2 macrophages is vital for inhibiting the progression of inflammation and facilitating the repair of liver cell damage in ALF [42, 60]. Figure 5a illustrates the operational workflow for validating the repolarization effects of FAND^HP@FuEVs^ at the cellular level. CLSM images, along with fluorescence intensity analysis of the two macrophage populations, demonstrated that, with increasing treatment duration, the proportion of M2 macrophages increased, whereas that of M1 macrophages decreased (Figure 5b, c). These data suggested that FAND^HP@FuEVs^ could facilitate the repolarization of M1 macrophages. Subsequently, flow cytometry was employed to quantitatively assess polarization outcomes. Treatment with various components led to a reduction in the expression of M1 macrophage and an increase in M2 macrophage expression (Figure 5d). The expression levels of the macrophage markers CD80 (M1) and CD206 (M2) are shown in Figure S13. The observed variations in the CD86/CD206 and CD80/CD206 ratios may be attributed to differences in macrophage polarization dynamics and the distinct biological roles of individual M1 markers, particularly CD80, which can exhibit context‐dependent expression patterns. Nevertheless, FAND^HP@FuEVs^ consistently promoted a shift away from the pro‐inflammatory M1 phenotype, as evidenced by the overall reduction in M1‐associated markers. To further demonstrate the pro‐repolarization effect of FAND^HP@FuEVs^, qRT‐PCR was conducted to evaluate the expression levels of canonical M1 (Cd86, Nos2) and M2 (Mrc1, Arg1) marker genes across all treatment groups. As shown in Figure S14, FAND^HP@FuEVs^ administration significantly upregulated M2‐related transcripts while suppressing M1‐associated gene expression. Furthermore, ELISA results indicated that FAND^HP@FuEVs^ facilitated the expression of the M2‐associated anti‐inflammatory factor IL‐10 and its marker protein Arg‐1 (Figure 5e, f). These findings further support that bioactive FAND^HP@FuEVs^ effectively promote the repolarization of M1 macrophages to M2 macrophages, laying a solid foundation for modulating the inflammatory microenvironment and exerting anti‐inflammatory effects.

In addition, a cell scratch assay was conducted to validate the regenerative effects of FAND^HP@FuEVs^ in treating ALF‐induced hepatocyte apoptosis. As shown in Figures 5 g, h and S15, the scratch area in the FAND^HP@FuEVs^ treatment group was significantly reduced compared to the PBS treatment group, with nearly complete closure observed after 24 h. This indicated the remarkable capability of FAND^HP@FuEVs^ to promote hepatocyte regeneration, attributable to the synergistic therapeutic effects of their components (HGF, PPC, and FuEVs). Collectively, these results demonstrate that the bioactive FAND^HP@FuEVs^, via the synergistic effects of their components, exhibit outstanding repolarization properties and facilitate the regeneration and repair of damaged hepatocytes, suggesting a promising in vitro therapeutic effect for ALF.

### 2.6 Restoration of Liver Function in Acetaminophen (APAP)‐Induced ALF Models

**FIGURE 6:**
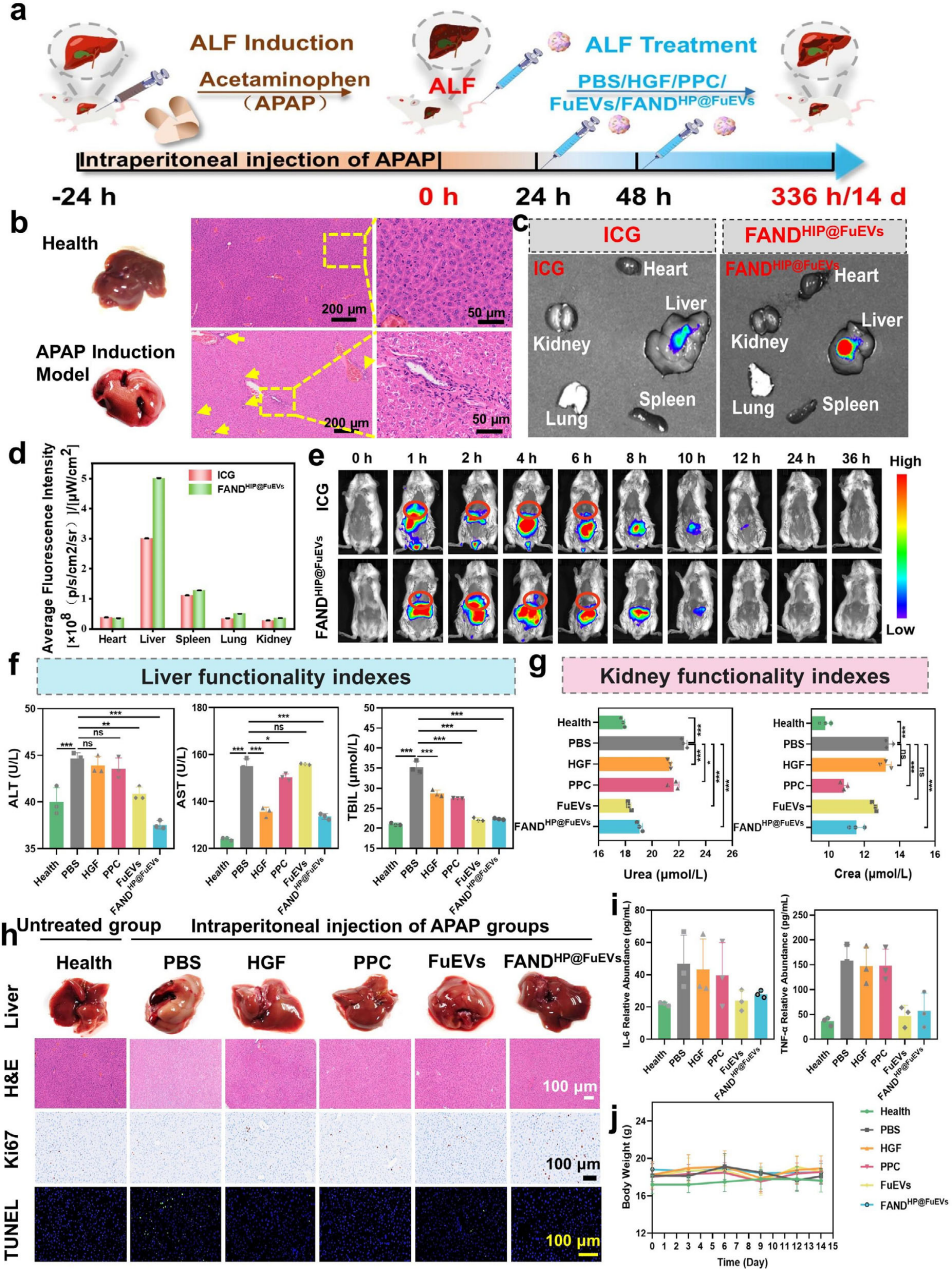
Restoration of liver function in acetaminophen (APAP)‐induced ALF models. (a) Schematic of the construction and treatment in the mouse ALF model induced by APAP; (b) representative images of healthy and ALF model mouse livers, along with H&E‑stained sections; (c) distribution; (d) quantification of fluorescence intensity in major organs 2 h after injection of free ICG and FAND^HP@FuEVs^ in the APAP‐induced ALF mouse model; (e) in vivo distribution of free ICG and FAND^HP@FuEVs^ in ALF model mouse over 36 h; (f) Serum levels of liver function markers AST, ALT, and TBIL (normal ranges: AST: 15–120 U/L, ALT: 5–40 U/L, TBIL: 1.7‐17 µmol/L); and (g) renal function markers Urea and Crea in different treatment groups (healthy, model, HGF, PPC, FuEVs, and FAND^HP@FuEVs^); (h) representative images of liver sections stained with H&E, Ki67, and TUNEL after various treatments; (i) changes in serum IL‑6 and TNF‑α levels in ALF mice following different treatments; (j) body weight changes across treatment groups. *p < 0.05; **p < 0.01; ***p < 0.001.

Hepatocyte damage and necrosis caused by drug abuse are critical factors in the onset of ALF [39, 40, 41]. To further validate the in vivo therapeutic effects of FAND^HP@FuEVs^, a mouse model of ALF was induced through intraperitoneal injection of APAP (350 mg kg^−1^) (Figure 6a). After 24 h, liver morphology was examined by H&E staining in both healthy and model mice, and serum ALT and AST levels were measured for comparison. As shown in Figure 6b and Figure S16, the livers of model mice exhibited a markedly roughened surface compared with the smooth, intact appearance of livers from control mice. Histological analysis by H&E staining revealed severe hepatocellular injury, characterized by nuclear pyknosis and fragmentation, as well as pronounced cytoplasmic vacuolization. Additionally, the levels of ALT and AST were significantly elevated, confirming the successful establishment of the ALF model. Indocyanine green (ICG) has been extensively employed in preclinical research to validate targeted drug delivery and to support therapeutic investigations in inflammatory diseases, particularly those affecting the liver [61, 62]. Subsequently, the targeting effect of FAND^HP@FuEVs^ on damaged liver tissue was verified with the fluorescent dye ICG. The ex vivo organ imaging and fluorescence intensity measurements demonstrated that FAND^HP@FuEVs^ preferentially accumulated in the inflamed liver. Moreover, in vivo imaging indicated that, compared with free ICG, FAND^HP@FuEVs^ rapidly and efficiently accumulated in the liver within 2 h post‑injection (Figure 6c–e), exhibiting strong in vivo targeting of inflammation.

After successfully targeting the liver, the therapeutic effects of various components (PBS, HGF, PPC, FuEVs, and FAND^HP@FuEVs^) on ALF were investigated. As shown in Figure 6f, g, the improvement of liver function markers (ALT, AST, and TBIL), as well as renal function indicators (urea and creatinine (Crea)), demonstrated that FAND^HP@FuEVs^ accelerated functional restoration in injured livers, indicating enhanced repair efficacy. Notably, as serum biomarkers ALT, AST, and TBIL exhibit dynamic changes during liver injury and recovery, their return to and maintenance within normal physiological ranges strongly indicate effective anti‐inflammatory activity and successful tissue repair. To further corroborate these functional improvements at the tissue and cellular levels, we next evaluated liver morphology and histopathological features and the results of H&E, Ki67, and TUNEL staining after 14 days of treatment. As shown in Figure 6h, compared with the PBS group, all treatment groups exhibited enhanced cytoarchitectural integrity, increased numbers of regenerating cells (Figure S17a), and decreased apoptotic cells (Figure S17b), indicating the significant role of FAND^HP@FuEVs^ in promoting hepatocyte regeneration and repair through their pleiotropic effects. In addition, serum levels of inflammatory factors IL‐6 and TNF‐α were measured after 14 days of treatment (Figure 6i), further confirming the reasonable in vivo anti‐inflammatory effects of FAND^HP@FuEVs^. Notably, both FuEVs and FAND^HP@FuEVs^ significantly suppressed pro‐inflammatory cytokines (IL‐6 and TNF‐α) in vivo. This shared anti‐inflammatory effect likely stems from the core functional role of the FuEVs component within the FAND^HP@FuEVs^ structure. As the foundational element of the formulation, FuEVs confer targeted immunomodulatory activity that drives cytokine downregulation, thereby accounting for the observed therapeutic overlap between the two treatment groups. Finally, although a mild reduction in body weight was observed immediately following disease induction, consistent with known compensatory responses in acute liver injury, such as fluid retention and metabolic adaptation [63], no significant changes in body weight were noted in any treatment group throughout the 14‐day therapeutic period (Figure 6j). This stability underscores the excellent biocompatibility of all formulation components. Additionally, a high survival rate in mice from all treatment groups was observed (Figure S18), indicating the favorable biosafety and therapeutic reparative effects of FAND^HP@FuEVs^. These results collectively demonstrate that, under the synergistic effects of HGF, PPC, and FuEVs, FAND^HP@FuEVs^ effectively target the liver in APAP‐induced ALF mice, exerting anti‐inflammatory, regenerative, and damage‐repairing actions. This strongly supports the promising therapeutic efficacy of the quadruplex bioactive FAND^HP@FuEVs^ in vivo.

### 2.7 Restoration of Liver Function in Hepatectomy‐Induced ALF Models

**FIGURE 7:**
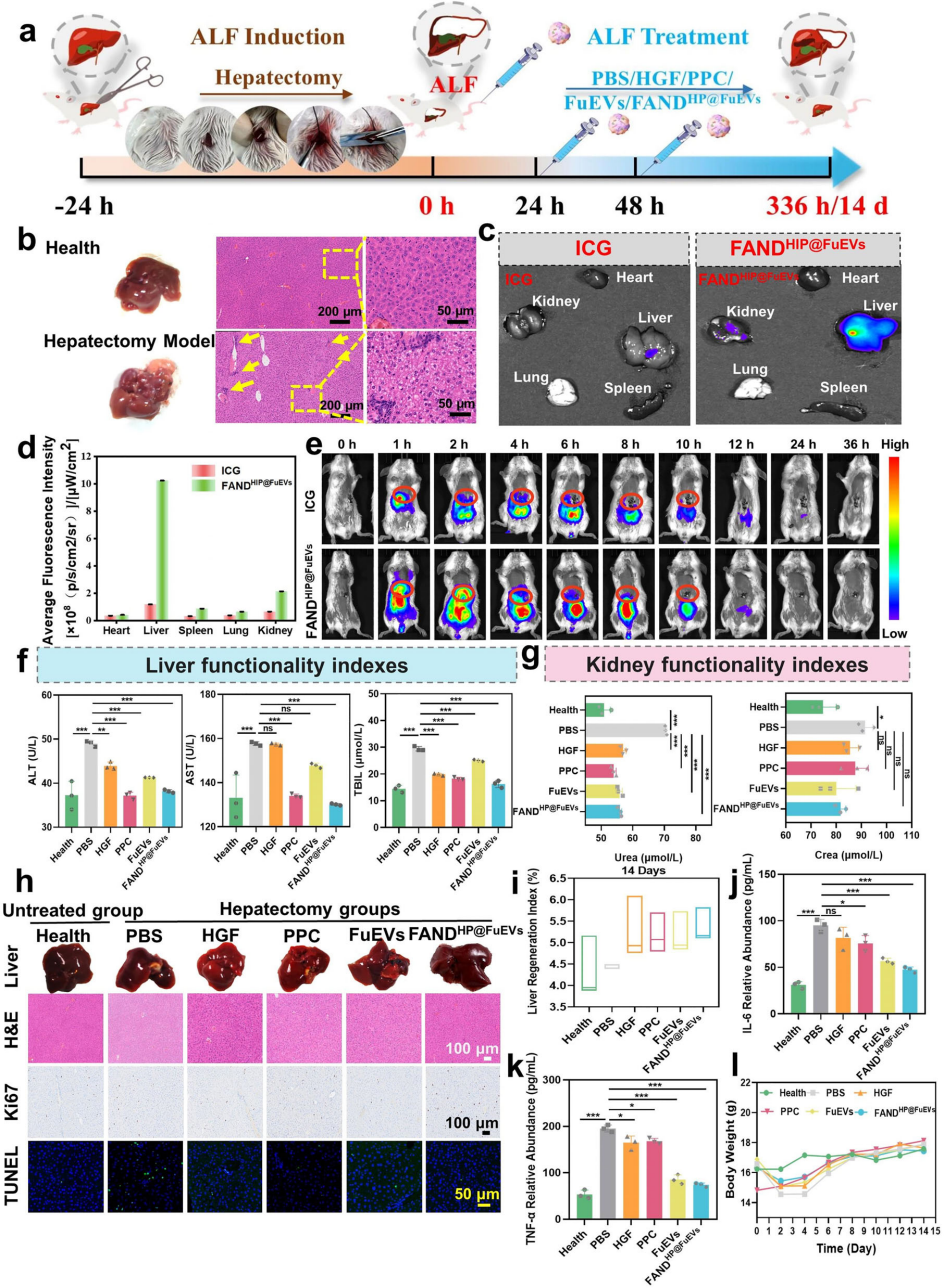
Restoration of liver function in hepatectomy‐induced ALF models. (a) Schematic of construction and treatment in the mouse ALF model induced by hepatectomy; (b) representative images of normal and ALF model mouse livers, along with H&E‑stained sections; (c) distribution; (d) quantification of fluorescence intensity in major organs 2 h after injection of free ICG and FAND^HP@FuEVs^ in the hepatectomy‐induced ALF mouse model; (e) in vivo distribution of free ICG and FAND^HP@FuEVs^ in ALF model mouse over 36 h; (f) Serum levels of liver function markers AST, ALT, and TBIL (normal ranges: AST: 15–120 U/L, ALT: 5–40 U/L, TBIL: 1.7–17 µmol/L); and (g) renal function markers Urea and Crea in different treatment groups (healthy, model, HGF, PPC, FuEVs, and FAND^HP@FuEVs^); (h) representative images of liver sections stained with H&E, Ki67, and TUNEL after various treatments; (i) liver regeneration index of different treatment groups; (j) changes in serum IL‑6 and TNF‑α levels in ALF mice following different treatments; (k) body weight changes across treatment groups. *p < 0.05; **p < 0.01; ***p < 0.001.

Inspired by the outstanding in vitro and in vivo therapeutic effects of FAND^HP@FuEVs^ on ALF, another in vivo study was further conducted using a mouse model of ALF induced by hepatectomy [64, 65, 66]. Initially, two‐thirds of the liver was resected to induce ALF, following clinical surgical procedures (Figure 7a). After 24 h, mice in both the model and healthy groups were euthanized and dissected to observe liver morphology and assess liver function indicators (ALT and AST). As shown in Figures 7b and S19, model mouse livers exhibited significant ischemia compared with those of the healthy controls. Corresponding H&E staining revealed extensive hepatocellular damage characterized by nuclear pyknosis and cytoplasmic vacuolization. These characteristic pathological alterations, combined with significantly elevated ALT/AST levels, clearly confirmed successful ALF model establishment. Subsequently, ex vivo organ imaging and fluorescence intensity data demonstrated significant accumulation of FAND^HP@FuEVs^ in the injured liver (Figure 7c,d). In addition, in vivo imaging further indicated that FAND^HP@FuEVs^ could efficiently accumulate in the liver (Figure 7e), laying the foundation for the safe and effective in vivo treatment of ALF.

Similarly, after 14 days of treatment, changes in liver and kidney function indicators (AST, ALT, TBIL, Urea, and Crea) were assessed (Figure 7f, g). Compared with the PBS group, FAND^HP@FuEVs^ treatment resulted in significant improvements in liver and kidney repair. Furthermore, the liver morphology of mice in the treatment groups showed marked differences, with the liver of the FAND^HP@FuEVs^ treatment group appearing more vibrant and well‐perfused than that in the model group (Figure 7h). Additionally, H&E staining of liver sections from the FAND^HP@FuEVs^‑treated group revealed reduced inflammatory cell infiltration (Figure 7h). Besides, Ki67 staining indicated that FAND^HP@FuEVs^ enhanced hepatocyte proliferation (Figure S20a), which is critical for liver regeneration, while TUNEL staining suggested that FAND^HP@FuEVs^ inhibited hepatocyte necrosis and facilitated liver damage repair (Figure S20b). Beyond that, the liver regeneration index serves as an important indicator for assessing hepatocyte growth. As shown in Figure 7i, FAND^HP@FuEVs^ and each component exhibited excellent hepatocyte growth‐promoting effects, with FAND^HP@FuEVs^ showing the most pronounced regenerative effect. Besides, changes in serum levels of the inflammatory factors IL‑6 and TNF‑α were validated across the treatment groups (Figure 7j, k), indicating the anti‐inflammatory effects of FAND^HP@FuEVs^ in vivo. Finally, during the 14‐day treatment period, there were no significant differences in body weight among the treatment groups (Figure 7l), further confirming the safety and efficacy of FAND^HP@FuEVs^ in promoting regenerative repair. Furthermore, survival analysis (Figure S21) revealed the favorable biosafety and therapeutic efficacy of FAND^HP@FuEVs^. The above results demonstrate that bioactive FAND^HP@FuEVs^ preferentially accumulate in the damaged liver in the hepatectomy‐induced ALF model, exhibiting outstanding anti‐inflammatory effects, promoting hepatocyte regeneration, and facilitating the repair of liver and kidney damage, thereby highlighting their robust therapeutic efficacy against ALF in vivo.

### 2.8 Biosafety Evaluation of the FAND^HP@FuEVs^ in Healthy Mice

**FIGURE 8:**
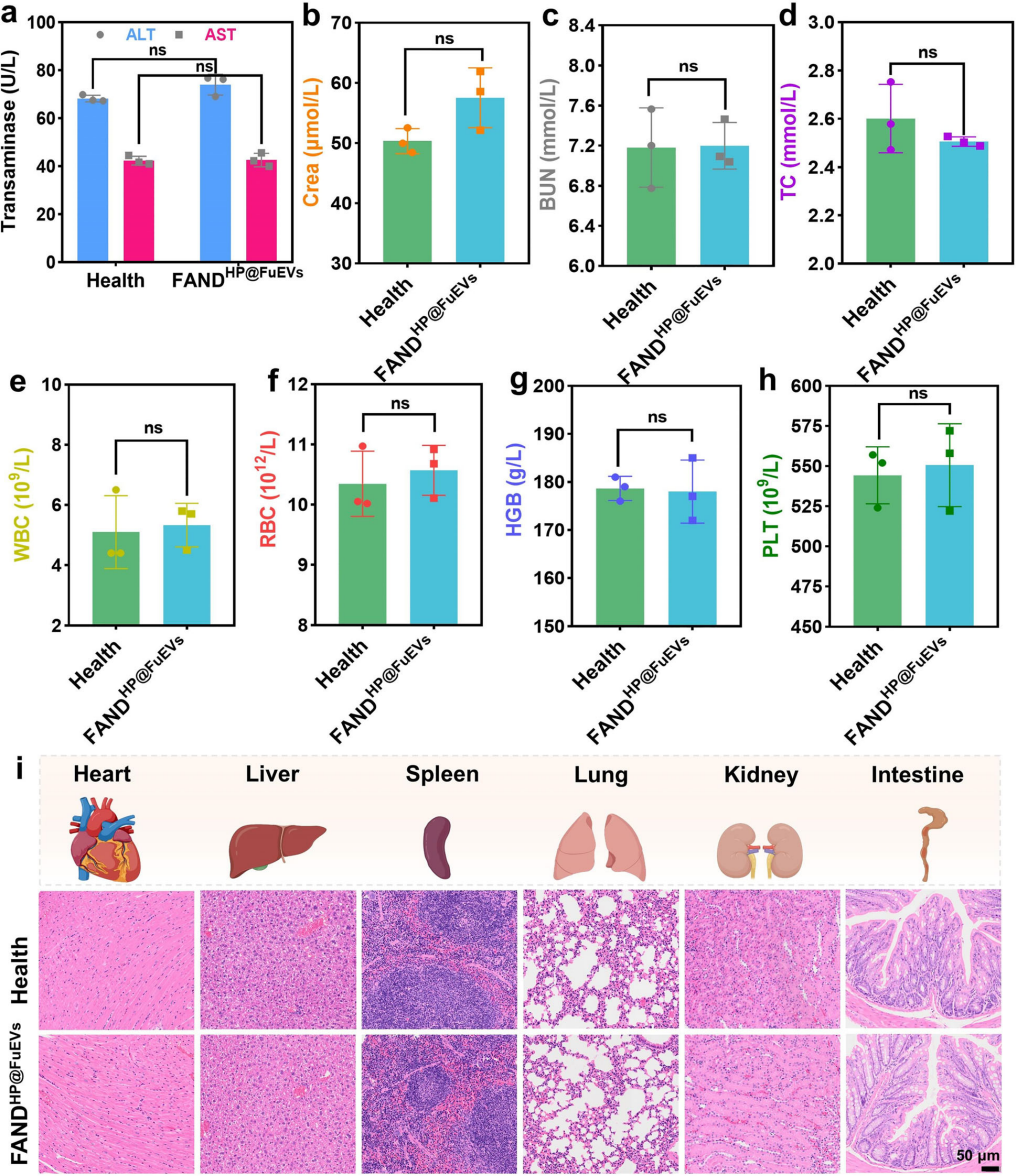
Biosafety assessment of FAND^HP@FuEVs^. Comparison of biochemical indicators (a) ALT and AST; (b) Crea; (c) BUN; (d) TC levels between healthy mice and the FAND^HP@FuEVs^ injection group. Comparison of hematological parameters; (e) WBC; (f) RBC; (g) HGB; (h) PLT counts between healthy mice and the FAND^HP@FuEVs^ injection group; and (i) H and E staining of heart, liver, spleen, lung, kidney, and intestine sections from the healthy group and the FAND^HP@FuEVs^ injection group.

Robust biocompatibility is essential for the clinical translation of nanotherapeutics. In the APAP‐ and hepatectomy‐induced ALF mouse models, comprehensive histopathological evaluation of major organs, including the heart, spleen, lung, kidney, and intestine, revealed no apparent tissue damage or pathological abnormalities in any treatment group (Health, PBS, HGF, PPC, FuEVs, or FAND^HP@FuEVs^) after 14 days of administration (Figure S22). These results, together with the observed therapeutic efficacy, confirm that FAND^HP@FuEVs^ exhibit both potent anti‐injury activity and excellent biosafety in diseased animals. To further assess systemic biocompatibility under non‐pathological conditions, FAND^HP@FuEVs^ were administered via tail vein injection in healthy mice, and no adverse effects were observed. As illustrated in Figure 8a–d, there were no notable differences in blood biochemical markers, including ALT, AST, Crea, blood urea nitrogen (BUN), and total cholesterol (TC), between the health control and the FAND^HP@FuEVs^ groups. Additionally, routine blood parameters such as white blood cell (WBC), red blood cell (RBC), hemoglobin (HGB), and platelet (PLT) showed no significant fluctuations, affirming the favorable biocompatibility of FAND^HP@FuEVs^ (Figure 8e–h). In addition, H&E staining of the heart, liver, spleen, lungs, kidneys, and intestines also revealed no significant lesions, further supporting the excellent biosafety of FAND^HP@FuEVs^ (Figure 8i). Furthermore, comparative analysis of serum complement activation markers (C3a, C5a, and soluble terminal complement complex sC5b‐9) among the healthy control (PBS), FAND^HP@FuEVs^‐treated group, and LPS‐induced inflammatory groups further confirmed the low immunogenicity and favorable long‐term safety profile of FAND^HP@FuEVs^ (Figure S23). These findings underscore their potential for effective ALF treatment and the broader therapeutic applications of bioactive FAND^HP@FuEVs^.

## 3 Conclusions

In conclusion, inspired by the recent concept of a FAND, we developed a quadruplex bioactive FAND^HP@FuEVs^ composed of two naturally derived EVs and two clinically approved biomacromolecules for treating both APAP‐ and hepatectomy‐induced ALF. Specifically, the FuEVs, formed by hybridizing M2‐EVs with MSC‐EVs, endowed the FAND^HP@FuEVs^ with dual intrinsic inflammation‐tropism capability, enabling targeted delivery to the injured liver site. Furthermore, the incorporated HGF and PPC, as therapeutic biomacromolecules clinically relevant for ALF treatment, substantially enhance the potential for clinical translation of FAND nanoformulations. Moreover, both in vitro and in vivo experiments demonstrated that the quadruple bioactive components in FAND^HP@FuEVs^—M2‐EVs, MSC‐EVs, HGF, and PPC—synergistically exhibited potent antioxidant, anti‐inflammatory, and macrophage repolarization effects while favorably promoting hepatocyte regeneration and repair. More importantly, the as‐established APAP‐ and hepatectomy‐induced ALF animal models, informed by clinical data from liver disease patients, display significant clinical relevance. In terms of the future development of FANDs, although the quadruplex bioactive formulation demonstrates promising therapeutic potential, several key aspects warrant further optimization. Particularly critical is the need to elucidate the underlying mechanisms of action and rigorously evaluate long‐term biosafety, two prerequisites for enabling robust clinical translation. Additional efforts should also focus on fine‐tuning the ratios of bioactive components to maximize therapeutic efficacy and establishing standardized, scalable protocols to facilitate the broader application of biomimetic FANDs. Overall, based on current findings and prospects, this work not only presents a fully bioactive nanodrug (FAND^HP@FuEVs^), extending the clinical applicability of EV‐ and biomacromolecule‐based nanodrugs, but also offers a safe, efficient, universal, and pleiotropic therapy for ALF and other inflammatory diseases.

## 4 Experimental Section

### 4.1 Patients

All samples used in this study were obtained with patient informed consent and in accordance with ethical guidelines. Written consent was obtained from all patients, and the study was approved by the Ethics Committee of the First Affiliated Hospital of Jinan University (KY‐2024‐117).

### 4.2 Animals

Adult female BALB/c mice (6–8 weeks old, 16–21 g) were purchased from Beijing Vital River Laboratory Animal Technology Co., Ltd. The animals were housed in standard laboratory barrier facilities with a 12/12 light‐dark cycle at 20–26°C and 50–60% relative humidity, with access to sufficient food and autoclaved water. Before the formal experiments, the mice could acclimate to the environment for 1–2 weeks. This study was conducted in strict accordance with the regulations for animal management and was supervised by the ethics committee of the Beijing Institute of Technology (BIT‐EC‐SCXK (Jing) 2021‐0011‐M‐127).

### 4.3 Cell Lines and Culture Conditions

Human umbilical cord mesenchymal stem cell lines (MSCs) and AML12 cell lines were purchased from Zhongqiao Xinzhou Biotechnology Co., Ltd. RAW264.7 cells were obtained from Shanghai Jinyuan Biotechnology Co., Ltd. AML12 cells were cultured in DMEM/F‐12 medium supplemented with 10% fetal bovine serum (FBS), 1% Penicillin‐Streptomycin solution, 1% insulin‐transferrin‐selenium (ITS), and 40 ng mL^−1^ dexamethasone in a humidified atmosphere with 5% CO2 at 37°C. MSCs were cultured in a specialized medium (Sciencell, 7501), while RAW264.7 cells were cultured in DMEM supplemented with 10% FBS and 1% Penicillin‐Streptomycin solution.

### 4.4 Reagents/Materials

The DMEM culture medium, DMEM/F‐12 culture medium, trypsin, and penicillin‐streptomycin were procured from M and C Gene Technology (Beijing, China). The specialized MSC medium and insulin‐transferrin‐selenium were obtained from SciClone (USA). The serum was purchased from PAN‐Biotech GmbH (Germany), and dexamethasone was sourced from Biosharp (China). Additionally, HGF, interleukin‐4 (IL‐4), and interleukin‐13 (IL‐13) were obtained from Pepro Tech (USA) (100‐39H‐25UG;214‐14‐50UG; 210‐13‐10UG) and Novoprotein (CJ72; CX56; CK74). Phosphatidylcholine, PEG4000, 2‐Methyl‐2‐butanol, and tribromoethanol were purchased from Macklin (Shanghai, China). ICG, reduced glutathione, and 4% paraformaldehyde were sourced from Aladdin (Shanghai, China). Furthermore, the 3‐(4,5‐Dimethylthiazol‐2‐yl)‐2,5‐diphenyltetrazolium bromide (MTT), BCA Kit, and ROS Probe (DCFH–DA) were all obtained from Solarbio Life Science (Beijing, China). The CCK‐8 kit was obtained from Biorigin (Beijing, China). The HGF ELISA kit and ELISA kit were purchased from NeobioScience (Shenzhen, China). The IL‐6 ELISA kit and TNF‐α ELISA kit were purchased from CLOUD‐CLONE CORP. (Wuhan, China). Interleukin‐10 (IL‐10) and arginase‐1(Arg‐1) assay kits were obtained from Elabscience (Wuhan, China). Additionally, PE/Cyanine7‐anti‐human CD63, PE‐anti‐human CD105, FITC‐anti‐human CD105, APC‐anti‐mouse CD206, FITC‐anti‐mouse CD206, APC‐anti‐mouse CD81, APC‐anti‐mouse CD106, APC‐anti‐mouse F4/80, TruStain FcXTM PLUS (anti‐mouse‐CD16/32), APC‐nti‐mouse CD80, FITC‐anti‐mouse CD80, and APC‐anti‐mouse CD86 were sourced from Pepro Tech (USA). Then, FastPure Cell/Tissue Total RNA Isolation Kit V2, HiScript III All‐in‐one RT SuperMix Perfect for qPCR, and Taq Pro Universal SYBR qPCR Master Mix were purchased from Vazyme Biotech Co., Ltd.

### 4.5 Polarization and Verification of M2 Macrophages

Prepare a culture medium containing 100 ng mL^−1^ of IL‐4 and 100 ng mL^−1^ of IL‐13. Transfer RAW264.7 (M0) cells into the medium with IL‐4 and IL‐13 and incubate them in a CO_2_ incubator for 24 h to polarize the cells, resulting in anti‐inflammatory M2 macrophages. Similarly, culture RAW264.7 cells in a medium with 1 µg mL^−1^ of LPS for 24 h to obtain pro‐inflammatory M1 macrophages. M2 macrophages were examined for the surface marker protein CD206 and M1 macrophages for the marker protein CD80 using laser confocal microscopy (CLSM). First, well‐maintained RAW264.7 cells were cultured in fluorescence confocal dishes with suitable ratios. The experimental groups included a blank control group (M0), LPS‐induced M1 cells, and IL‐4/IL‐13‐induced M2 cells. After polarization, a serum‐free medium was prepared with FITC‐anti‐mouse CD80 (1.5 µL) and APC‐anti‐mouse CD206 (1.5 µL) staining solution. Then, 1 mL of the staining solution was added to each dish, followed by incubation at 37°C for 30 min. Finally, CLSM was utilized to preliminarily verify the successful polarization of macrophages.

The levels of the M2 macrophage marker factors IL‐10 and Arg‐1, as well as the M1 macrophage markers IL‐6 and TNF‐α, were measured before and after polarization using ELISA to validate cell polarization. RAW264.7 cells were seeded in a 6‐well plate and polarized for 24 h. Following polarization, the cells were digested with trypsin to prepare a cell suspension. The cells were then sonicated and centrifuged, and the supernatant was collected to prepare samples for analysis. Taking the ELISA assay for the cytokine IL‐10 as an example, the steps are as follows. The washing, working solutions, and standard dilutions. Then, the samples underwent incubation and reactions for 90, 60, 30, and 15 min, respectively. Finally, the absorbance of the samples and standards was measured at 450 nm using a microplate reader. The same procedures apply to the detection of IL‐6, TNF‐α, and Arg‐1 using their respective ELISA kits.

Macrophage polarization was assessed using flow cytometry, focusing on the detection of the M2 macrophage marker protein CD206. First, collect the polarized M2 cells and the unpolarized control group (M0 cells). Replace the culture medium with cold PBS, ensuring a cell count of at least 10^5. Next, add 1 µL of non‐specific antibody‐blocking solution TruStain FcX PLUS (anti‐mouse‐CD16/32) to each tube and incubate on ice for 7 min. Then, add 50 µL of the membrane permeabilizing agent Triton X‐100 and incubate for 5 min. Centrifuge at 1000 rpm for 5 min, discard the supernatant, and resuspend the cells in PBS. Subsequently, add 2 µL of FITC‐anti‐mouse CD206 and APC‐anti‐mouse F4/80 to each tube and incubate on ice for 30 min. After incubation, centrifuge again to discard the supernatant, wash with PBS, and centrifuge to remove the supernatant. Finally, resuspend the cells in 400 µL of PBS for flow cytometric analysis. For M1 macrophages, after incubating with fluorescently labeled antibodies against CD80 (2 µL) and CD86 (2 µL), perform PBS washes before directly proceeding to detection.

### 4.6 Extraction and Characterization of M2‐EVs and MSC‐EVs

Using differential ultracentrifugation (300 g for 15 min, 2000 g for 15 min, 10,000 g for 30 min, 100,000 g for 70 min, and 120,000 g for 70 min at 4°C) to extract two types of EVs. Structural characterization of EVs includes TEM morphological analysis and particle size and potential characterization. TEM morphological characterization: Purified MSC‐EVs and M2‐EVs were diluted. A 10 µL drop of EVs was placed on a carbon‐coated copper grid and fixed for 2 min. Then, excess liquid was absorbed with filter paper along the edge of the grid. Next, 10 µL of uranyl acetate was applied to the grid and immediately absorbed. Repeat this step twice. After that, 10 µL of uranyl acetate was left to stain for 1 min, then dried with filter paper, allowing for direct observation using TEM. Additionally, particle size and potential characterization: MSC‐EVs and M2‐EVs were diluted with sterile triple‐distilled water. Following the manufacturer's instructions, NTA was conducted to assess the particle size distribution of the EVs. Simultaneously, the Malvern ZEN 3600 dynamic light scattering (DLS) analyzer was used to measure the zeta potential of the EVs.

The property characterization of EVs involves measuring protein concentration and detecting surface marker proteins. The BCA protein assay kit was used to measure protein concentration. Firstly, mix BCA reagents A and B in a 50:1 ratio to prepare the working solution. Then, the bovine serum albumin (BSA) was diluted to various concentrations for a standard curve, and then 20 µL of MSC‐EVs, M2‐EVs, and the BSA solutions were added to a 96‐well plate along with 200 µL of BCA working solution. After incubating at 37°C for 15–30 min, the absorbance at 562 nm was measured to create a standard curve for calculating the EV concentrations. For surface marker protein detection, MSC‐EVs and M2‐EVs were incubated with FITC‐anti‐human CD105 and APC‐anti‐mouse CD206 at 37°C for 30 min. The samples were transferred to a 100 kDa ultrafiltration tube and centrifuged at 8500 rpm, 4°C for 15 min to remove unbound antibodies. After cleaning, the stained EVs were placed on a glass slide, covered with a cover slip, and sealed. They were then observed under a laser confocal microscope using a 100× oil immersion objective to qualitatively assess the expression of CD105 and CD206 in the EVs.

The marker proteins CD105 for MSC‐EVs, CD206 for M2‐EVs, and CD63 and CD81 for the general surface markers were analyzed by flow cytometry. For the detection of CD206 and CD81 on M2‐EVs, a suitable amount of EVs was placed in a 1.5 mL centrifuge tube. Based on the concentration and particle number of the EVs, direct antibodies (PE anti‐mouse CD81 (1.2 µL) and APC‐anti‐mouse CD206 (2 µL)) were added and incubated on ice for 30 min, with controls set aside for blank and voltage adjustment. After incubation, the contents of the tube were transferred to a pre‐cooled 100 KD ultrafiltration tube, followed by the addition of sterile pre‐cooled PBS buffer. The mixture was centrifuged at 8500 rpm and 4°C for 10 min, with this washing step repeated 2–3 times to remove unbound antibodies. Finally, the labeled EVs were transferred into flow cytometry tubes and analyzed using flow cytometry.

### 4.7 Preparation of FuEVs

FuEVs were prepared by inducing the fusion of MSC‐EVs and M2‐EVs using PEG4000. First, NTA was used to determine the particle number of both types of EVs, ensuring they were similar. A total of 200 µL of each type of EV was combined in a 50 mL centrifuge tube at a 1:1 volume ratio. Gradually, 400 µL of PEG4000 solution, equal to the volume of the mixed EVs, was added dropwise to the tube. After gentle mixing, the solution was allowed to sit at room temperature for 10 min. Next, 4 mL of serum‐free pure medium, five times the total volume (800 µL), was added slowly over 2 min. An additional 12 mL was then added, making the total volume of pure medium 20 times that of the initial mixture. The liquid from the centrifuge tube was transferred to a culture dish and incubated at 37°C for 40 min. After incubation, the liquid was transferred to a 100 KD ultrafiltration tube and centrifuged at 8500 rpm for 10–15 min at 4°C. The retained liquid in the ultrafiltration tube was collected, yielding the FuEVs.

FRET analysis was employed to validate vesicle fusion. Dil‐labeled MSC‐EVs and DiD‐labeled M2‐EVs were subjected to PEG 4000‐mediated fusion post‐ultrafiltration, yielding FuEVs. A control mixture (Mix) containing both labeled EV types at matching ratios was prepared. Comparative fluorescence intensity profiles of Dil‐MSC‐EVs, DiD‐M2‐EVs, FuEVs, and the Mix group were analyzed.

### 4.8 Validation and Characterization of FuEVs

The successful fusion of FuEVs was verified using CLSM. Similar to the characterization of individual EVs, the validation of FuEVs required the simultaneous addition of fluorescently labeled antibodies: FITC‐anti‐human CD105 to label MSC‐EVs and APC‐anti‐mouse CD206 to label M2‐EVs. After centrifugation at 8500 rpm for 10 min at 4°C and washing twice, 5 µL of the sample was placed on a glass slide and covered with a coverslip. Observations were made under a 100× objective to examine the overlap of the two fluorescent signals. Single vesicles and mixed vesicles served as controls (MIX) to confirm the successful preparation of FuEVs. Subsequently, the protein concentration of FuEVs was measured using a BCA protein assay kit. TEM was employed to characterize the morphology of FuEVs, while NTA was used to measure particle size. The zeta potential was determined using a Malvern particle size analyzer.

### 4.9 Comparison of miRNA Sequencing Between FuEVs and Single Vesicles

This experiment includes small RNA sequencing and data analysis. First, the small RNA sequencing was performed using Illumina HiSeq/Novaseq or MGI2000 platforms. Library preparation was done using 1 µg total RNA or 10 ng small RNA. Then, the 3’ SR adapter for Illumina was ligated to the small RNA using a 3’ ligation enzyme. Next, excess 3' SR adapter was hybridized with the SR RT primer for Illumina to prevent adapter dimers. After that, the 5’ SR adapter for Illumina was ligated to the small RNA using a 5’ ligation enzyme, and the first‐strand cDNA was synthesized using ProtoScript II reverse transcriptase. Each sample was then amplified by PCR using P5 and P7 primers, and the PCR products were purified using DNA clean beads. The purified products, which were between 140 and 160 bp, were recovered and further cleaned up using PAGE and validated with an Agilent 2100 Bioanalyzer.

The data analysis involves several key steps: quality control, microRNA identification, differential expression analysis, GO and KEGG enrichment analysis, microRNA target mRNA analysis, and principal component analysis (PCA). Using the comparison of FuEVs and MSC‐EVs sequencing as an example, the detailed experimental procedure is outlined below. To eliminate technical sequences, FASTQ format data that passed the initial quality filter were processed using Trimmomatic (v0.30) to generate high‐quality clean data. The processing involved several steps: first, adapter sequences were removed; next, bases from the 5' or 3’ ends that contained “N” or had quality scores below 20 were trimmed. Following this, any remaining bases with an average quality score lower than 20 were discarded using a sliding window approach of 4 bp. Finally, reads shorter than 18 bp after trimming were removed. Subsequently, microRNAs and their expression data were identified using the miRDeep2 software. After this, differential expression analysis was conducted using edgeR. The microRNAs with a *p*‐value less than 0.05 and with a fold change greater than or equal to 1 were identified as differentially expressed. Additionally, GOSeq (v3.3a) was employed to identify GO terms associated with microRNA target genes, using a significance threshold of padj < 0.05. The significantly differentially expressed genes were enriched in KEGG pathways. Subsequently, Miranda was used to predict microRNA‐mRNA interactions. Finally, PCA was applied to simplify the data and eliminate secondary factors and noise.

### 4.10 Screening of PPC and HGF Concentrations

Before loading drugs using electroporation, it is essential to consider the potential cytotoxic effects of high drug concentrations. Therefore, a preliminary screening of drug concentrations is necessary. For PPC concentration screening, PPC was dissolved in a methanol‐water mixture at a ratio of 1:9, and subsequently, the culture medium was used to prepare PPC solutions at concentrations of 0, 2.5, 5, 10, 20, 50, 100, 200, and 500 µM. After preparation, the MTT assay was employed to assess the cytotoxicity of PPC on cells. The specific procedure involved seeding AML12 mouse liver cells at a density of 10^4 cells per well in a 96‐well plate and incubating them for 24 h. Following this, the fresh medium containing different concentrations of PPC was added to the wells. The corresponding control group received only the specialized medium. After incubation for 24 h, the medium in each well was discarded, and 10% MTT solution was added, followed by a 4 h dark incubation. Afterward, 110 µL of DMSO was added to each well to dissolve the resulting formazan crystals. After shaking the plate for 15 min in a microplate reader, the absorbance at 490 nm was measured. The absorbance values reflected cell viability and were used to select the appropriate PPC concentration. Meanwhile, HGF concentration optimization was performed using the same method. The lyophilized HGF standard was dissolved in a pure culture medium to prepare gradient solutions at concentrations of 0, 20, 50, 100, 200, and 400 ng ml^−1^. Different concentrations of HGF were mixed with the cells and incubated, followed by measuring the relevant MTT values to determine the impact of HGF on cell viability and to finalize the appropriate HGF concentration.

### 4.11 Construction of FAND^HP@FuEVs^

After screening of PPC and HGF concentrations, the selected appropriate concentrations of PPC (100 µM, 135 µL) and HGF (200 ng ml^−1^, 135 µL) were mixed in specific ratios and introduced into FuEVs (1.2 mg ml^−1^, 30 µL) using electroporation. Following a series of operations, including incubation and ultrafiltration, FAND^HP@FuEVs^ were obtained. The detailed procedure is as follows: First, the prepared solutions were mixed with FuEVs, and the mixture was transferred to an electroporation cuvette that had been pre‐chilled on ice. The parameters of the electroporator were set to 100 V, 200 Ω, and 100 µF. After electroporation, the sample from the cuvette was transferred to a sterile culture dish and placed in a CO_2_ incubator for 1 h to promote membrane repair. Following the incubation, the liquid in the culture dish was transferred to a 100 KD ultrafiltration tube and centrifuged at 8500 rpm for 10 min at 4°C to remove any free drug. Finally, the ultrafiltered sample collected from the ultrafiltration tube yielded FAND^HP@FuEVs^.

### 4.12 Validation and Characterization of FAND^HP@FuEVs^

The drug loading in FAND^HP@FuEVs^ was verified using UV absorption directly. First, different PPC concentrations (500, 250, 125, 62.5, 31.3, and 15.6 µM) were transferred to UV spectrophotometry cuvettes, and the absorbance of these samples was measured. Simultaneously, FAND^HP@FuEVs^ were diluted appropriately and measured under the same baseline conditions. The standard absorption curve was established based on the absorbance values of the PPC solutions, allowing calculation of the PPC loading in the FAND^HP@FuEVs^. In addition, for the determination of HGF loading, the ELISA method was employed. Following the instructions of the human HGF ELISA kit, the HGF loading in the FAND^HP@FuEVs^ was calculated based on the HGF concentration standard curve.

The properties were primarily assessed using a BCA assay kit to determine protein concentration. Structural characterization included examining the morphology, particle size, and zeta potential of the drug delivery system via TEM. Additionally, the stability of the drug delivery system over 7 days was evaluated. For stability assessment, frozen drug‐loaded vesicles were thawed on ice, and the particle size and zeta potential were measured at the same time points over 7 consecutive days. The data were presented as a variation curve to illustrate the dispersion stability of the materials.

### 4.13 Cell Uptake Analysis

CLSM was used to evaluate cell uptake of FAND^HP@FuEVs^. First, AML12 (10^5 mL^−1^) was seeded in confocal fluorescence plates and cultured overnight. Based on the amount of FuEVs in the drug‐loaded system, specific quantities of FAND^HP@FuEVs^ and FuEVs were incubated with FITC‐anti‐human CD105 (5 µL) and APC‐anti‐mouse CD206 (2 µL) in a 37°C incubator for 30 min. After incubation, the samples were transferred to 100 KD ultrafiltration tubes, mixed with PBS, and centrifuged at 8500 rpm for 15 min at 4°C, repeating this process 3 times. After centrifugation, the antibody‐labeled samples were cultured with AML12 for 4 h. After washing 2 times with sterile PBS, the uptake was determined by CLSM.

Meanwhile, ICG (100 µg mL^−1^, 80 µL)‐loaded FAND^HIP@FuEVs^ were prepared, and CLSM was used to observe the uptake of FAND^HIP@FuEVs^ and free ICG by cells. AML12 was seeded at a density of 10^5 mL^−1^ in confocal fluorescence plates and cultured overnight. The concentration of free ICG was determined based on the amount of ICG in the materials. Appropriate amounts of FAND^HIP@FuEVs^ and ICG were diluted and incubated with AML12 for 4 h. After incubation, the cells were washed 3 times with sterile PBS and observed under CLSM for comparative analysis.

Furthermore, M2‐EVs, MSC‐EVs, and FuEVs were labeled with DiO dye. After ultrafiltration to isolate the labeled vesicles, they were incubated with both normal and inflammatory cells for 4 h. PBS‐treated groups served as controls to assess cellular uptake of each vesicle type.

### 4.14 Cytotoxicity Analysis

Using FAND^HP@FuEVs^ to assess cytotoxicity in RAW264.7 cells and AML12 cells, the procedure was as follows: First, RAW264.7 cells were seeded at a density of 10^4 cells per well in a 96‐well plate and cultured for 24 h. After that, FAND^HP@FuEVs^ were diluted in RAW264.7‐specific culture medium to various concentrations: 0, 2.5, 3.125, 5, 6.25, 10, 12.5, 20, 25, and 40 µg ml^−1^. These different concentrations were added to the 96‐well plate, along with a blank control group containing only cell culture medium. The plates were incubated for an additional 24 h. After incubation, the liquid in each well was removed, and 10% MTT solution was added for a 4 h dark incubation. Following this, 110 µL of DMSO was quickly added to each well to dissolve the formed formazan crystals. Finally, the parameters of the microplate reader were set to measure the absorbance at 490 nm after shaking and incubating at 37°C for 15 min. The absorbance values indicated the level of cell viability, which was used to analyze the cytotoxic effects of each component on the cells.

### 4.15 Analysis of Cell Proliferation

First, a straight ruler and marker were placed under UV light for 30 min. Using these tools, parallel lines were drawn evenly across the bottom of a 6‐well plate, ensuring at least five lines per well. Then, AML12 cells were seeded at a density of 2 × 10^5 in the 6‐well plate and cultured for 24–48 h until they covered the bottom of the plate. At this point, a 10 µL pipette tip was used to create scratches vertically across the plate, followed by a gentle wash with sterile PBS to remove cell debris. FAND^HP@FuEVs^, FuEVs, HGF, and PPC were diluted to appropriate concentrations in a specific cell culture medium and added to the 6‐well plate, with a control group containing only culture medium without any additives. The plates were incubated in a CO_2_ incubator, and images were taken at 0, 12, and 24 h using a microscope to preliminarily assess cell proliferation. To further quantify the area changes of the scratches, the experiment was repeated using CLSM, and ImageJ software was employed for quantitative analysis of the scratch area changes.

### 4.16 Analysis of Antioxidant Stress

To determine the half‐maximal lethal concentration (LC50) of H_2_O_2_ on cells, various concentrations of H_2_O_2_ solutions were prepared using cell culture media: 0, 50, 100, 200, 250, 400, 500, 800, 1000, and 2000 µM. These were then incubated with AML12 cells in a 96‐well plate for 24 h. Following this, MTT solution was added and incubated for 4 h, after which DMSO was added to dissolve the crystals, and absorbance was measured at 490 nm to determine the LC50 of H_2_O_2_.

For the fluorescence quantification of ROS, AML12 cells were seeded at a density of 10^5 in fluorescence plates and cultured overnight. Then, a solution of 800 µM H_2_O_2_ was added for 24 h. After incubation, appropriate amounts of PPC, HGF, FuEVs, and FAND^HP@FuEVs^ were added, along with a blank control group and a positive control group containing only H_2_O_2_. After an additional 24 h incubation, a ROS probe (DCFH–DA) diluted in serum‐free media was added and incubated at 37°C in the dark for 30 min. After washing with sterile PBS, observations were made using a laser confocal microscope.

For the MTT analysis of antioxidant capacity, AML12 cells were seeded at a density of 10^5 in a 96‐well plate and cultured. Following the incubation protocol described above, 800 µM H_2_O_2_ was added and incubated for 24 h. After that, the media was replaced with appropriate amounts of PPC, HGF, FuEVs, and FAND^HP@FuEVs^. After another 24 h of incubation, MTT solution was added, and DMSO was used to dissolve the crystals. The absorbance at 490 nm was measured to calculate cell viability and analyze the anti‐inflammatory and antioxidant stress capabilities of the materials and components on AML12 cells.

### 4.17 Analysis of Macrophage Repolarization

CLSM, flow cytometry quantification, and ELISA to assess changes in cytokine levels were utilized to analyze the repolarization effect. For CLSM, RAW264.7 cells were first seeded in fluorescence plates. One group was supplemented with media containing IL‐4 (100 ng mL^−1^) and IL‐13 (100 ng mL^−1^), while the remaining three groups received media containing 1 µg ml^−1^ of LPS, and the M0 group served as a blank control. After 24 h of incubation, the media from the M2, one group of M1, and the M0 cells were removed, and FITC‐anti‐mouse CD206 and APC anti‐mouse CD81 were added for a 30‐minute incubation. The old media from the two M1 cell groups were replaced with an equal amount of FAND^HP@FuEVs^, and they were cultured further in the incubator. After antibody incubation, cells were gently washed with sterile PBS, observed under CLSM, and the corresponding fluorescence intensity values were recorded. This process was repeated every 24 h for 48 h, observing one group of M1 cells each time with the two fluorescent antibodies and recording fluorescence intensity for comparison. Flow cytometry quantification and ELISA for cytokine level changes were performed as described above.

qRT‐PCR validation of FAND‐induced macrophage repolarization: Total RNA was extracted from treated cell groups using a FastPure Cell/Tissue Total RNA Isolation Kit V2 (Catalog No. 017E2203KA, Vazyme Biotech Co., Ltd.) following the manufacturer's protocol. Complementary DNA (cDNA) was synthesized through reverse transcription with a HiScript III All‐in‐one RT SuperMix Perfect for qPCR (Vazyme Biotech Co., Ltd.). Real‐time PCR was subsequently performed using a Taq Pro Universal SYBR qPCR Master Mix (Vazyme Biotech Co., Ltd.) on the Applied Biosystems 7500 Real‐Time PCR System. Gene expression levels of M1/M2 polarization markers (CD86, iNOS for M1; CD206, Arg‐1 for M2) were quantified relative to GAPDH using the 2‐ΔΔCt method.

### 4.18 Evaluation of the Targeting Ability and Imaging Effects of FAND^HP@FuEVs^

Model mice were randomly divided into two groups, receiving injections of ICG and FAND^HIP@FuEVs^ via the tail vein. Afterward, in vivo imaging of the mice was performed every two hours. Based on the in vivo fluorescence imaging results, ex vivo imaging of the organs (heart, liver, spleen, lung, and kidney) was conducted using a small animal imaging system, followed by quantitative fluorescence analysis to comprehensively evaluate the imaging performance and targeting ability of ICG and FAND^HIP@FuEVs^. The evaluation of the targeting ability and imaging effects of FAND^HP@FuEVs^ in the APAP‐ and hepatectomy‐induced ALF mice followed the same procedures.

### 4.19 Construction and Validation of an APAP‐induced ALF Model in Mice

Female mice (6–8 weeks) were acclimated in a standard laboratory environment for 1–2 weeks, with adequate food and water provided. The body weight of the mice was recorded to determine the appropriate APAP dosage of 300 mg/kg. APAP was dissolved in physiological saline and administered via intraperitoneal injection. After 24 h, paraffin embedding was performed to prepare H and E‐stained sections of the liver tissue for observation of changes, validating the success of the model construction.

### 4.20 In Vivo Therapeutic Effects Evaluation of FAND^HP@FuEVs^

First, PBS, HGF, PPC, FuEVs, and FAND^HP@FuEVs^ were injected into the mice via the tail vein. After 14 days of therapy, blood was collected to measure the biochemical parameters and hematological parameters of the different groups. Meanwhile, ELISA was used to determine the levels of inflammatory factors to assess the levels of inflammation and liver damage in the ALF mice. The evaluation of the therapeutic effects of FAND^HP@FuEVs^ in the hepatectomy‐induced ALF mice followed the same procedures.

### 4.21 Liver Morphology and Histological Observation

After 14 day‐therapy, the livers from different groups were harvested and photographed for observation. The liver tissue was then fixed in 4% paraformaldehyde. All samples were collected to prepare H and E‐stained sections, Ki67‐stained sections, and TUNEL‐stained sections. The sections were observed under a microscope, and the results were used to assess the in vivo therapeutic effects of FAND^HP@FuEVs^. The evaluation of the therapeutic effects of FAND^HP@FuEVs^ in the hepatectomy‐induced ALF mice followed the same procedures.

### 4.22 Construction and Validation of Hepatectomy ALF Model in Mice

Before liver resection surgery, the mice were anesthetized and fixed on the surgical table. After disinfection with alcohol, a small incision was made along the midline of the mouse's abdomen using surgical scissors. Two fingers were used to press against the abdominal wall while the other hand gently pulled the liver lobules out. Using flat tweezers, the liver lobules were carefully grasped and pulled, followed by multiple ligations with surgical sutures at the base of the lobule for hemostasis. The ligated liver lobules were then cut off, and this process was repeated for other liver lobes, resulting in approximately two‐thirds of the entire liver being resected. After resection, the incision was carefully sutured, and an appropriate amount of antibiotics and physiological saline was injected into the abdominal cavity to prevent infection. 24 h after modeling, healthy and model mice were euthanized, and the harvested livers were fixed in 4% paraformaldehyde. Subsequently, paraffin embedding was performed to prepare H&E‐stained sections of the liver tissue for observation of changes, validating the success of the model construction.

### 4.23 Liver Regeneration Index Evaluation

After 14 day‐therapy, the liver regeneration index for different groups of mice was assessed using the following formula: liver regeneration index = (*B*/*G*) × 100%, where *B* represents the weight of the regenerated liver and *G* represents the body weight.

### 4.24 Biosecurity Estimations

Normal mice were injected with FAND^HP@FuEVs^ and fed for 14 days. Blood biochemical parameters and hematological parameters were measured to verify the biosafety of FAND^HP@FuEVs^. Additionally, H and E‐stained tissue sections of the heart, spleen, lung, kidney, and intestine from both groups of mice were prepared for comparative observation. Furthermore, comparative analysis of serum complement activation markers (C3a, C5a, and sC5b‐9) among the healthy control group (PBS), the FAND‐treated group, and the LPS‐induced inflammatory group to confirm the low immunogenicity and favorable long‐term safety of FAND^HP@FuEVs^.

### 4.25 Statistical Analysis

All data were presented as mean ± standard deviation (SD). GraphPad Prism 8 software (GraphPad Software Inc., San Diego, CA, USA) was used for statistical analyses. Comparison between two or multiple groups was conducted using Student's unpaired *t*‐test or one‐way analysis of variance (ANOVA). *P* values < 0.05 were considered statistically significant (*p < 0.05; **p < 0.01; ***p < 0.001).

## Author Contributions

J. Zhang and M. Hu designed and conceived this study. M. Sun carried out all the experiments. M. Sun, F. Fang, J. Liu, Y. Fan, S. Wang and J. Zhang contributed to data collection and interpretation. M. Sun wrote the manuscript. J. Zhang, M. Hu, J. Liu, C. Zhang, W. Li, Z. Quan and D. Zhao reviewed and helped shape the research, analysis, and manuscript. J. Zhang, M. Hu and J. Liu contributed to Funding acquisition. All the authors revised the manuscript.

## Ethics Statement

Patients: All samples used in this study were obtained with patient informed consent and ethical guidelines. Written consent was obtained from all patients, and the study was approved by the Ethics Committee of the First Affiliated Hospital of Jinan University (KY‐2024‐117).

Animals: Adult female BALB/c mice (6‐8 weeks old, 16–21 g) were purchased from Beijing Vital River Laboratory Animal Technology Co., Ltd. The animals were housed in standard laboratory barrier facilities with a 12/12 light‐dark cycle at 20–26°C and 50–60% relative humidity, with access to sufficient food and autoclaved water. Before the formal experiments, the mice could acclimate to the environment for 1–2 weeks. This study was conducted in strict accordance with the regulations for animal management and was supervised by the ethics committee of the Beijing Institute of Technology (BIT‐EC‐SCXK (Jing) 2021‐0011‐M‐127).

## Conflicts of Interest

The authors declare no conflicts of interest. Jinfeng Zhang is a member of the *Exploration* editorial board, and she was not involved in the handling or peer review process of this manuscript.

## AI Statement

During the preparation of this work, the authors used ChatGPT (GPT‑4o) only for language editing and grammar checking to improve the readability and clarity of the manuscript. No AI tools were used for scientific data generation, experimental design, data analysis, figure preparation, or the formulation of conclusions. After using the tool, the authors carefully reviewed and revised the content as necessary and take full responsibility for the content of the publication.

## Supporting information




**Supporting File**: exp270204‐sup‐0001‐SuppMat.docx.

## Data Availability

The data that support the findings of this study are available from the corresponding author upon reasonable request.
