# Supplementary material for: Quadruplex Bioactive FAND for Treating Acute Liver Failure Induced by Acetaminophen or Hepatectomy
Source: Exploration (Beijing). 2026 Jul 31:70204. Online ahead of print. doi: 10.1002/exp2.70204 (PMC13426876; doi:10.1002/exp2.70204)
Supplement: Supplementary file 1 — Supporting File: exp270204‐sup‐0001‐SuppMat.docx. [file EXP2-9999-0-s001.docx]

Supplementary Information

Quadruplex Bioactive FAND for Treating Acute Liver Failure Induced by Acetaminophen or Hepatectomy

**
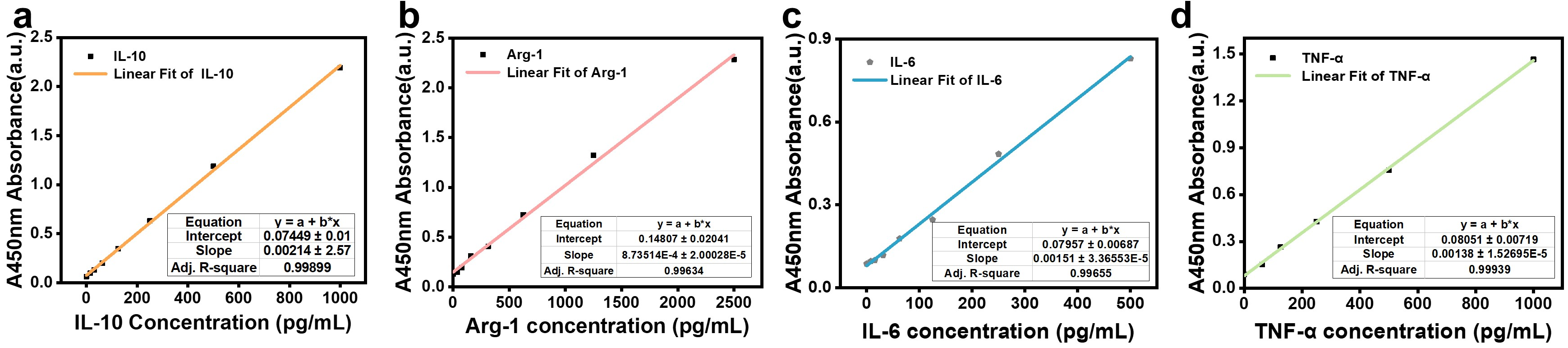
**

**Figure S1. The standard absorption curves for the concentrations of cytokine interleukin-10 (IL-10), arginase-1 (Arg-1), cytokine interleukin-6 (IL-6), and tumor necrosis factor-alpha (TNF-α).** (a) The standard absorption curve for interleukin-10 (IL-10). (b) The standard absorption curve for arginase-1 (Arg-1). (c) The standard absorption curve for interleukin-6 (IL-6). (d) The standard absorption curve for tumor necrosis factor-alpha (TNF-α).


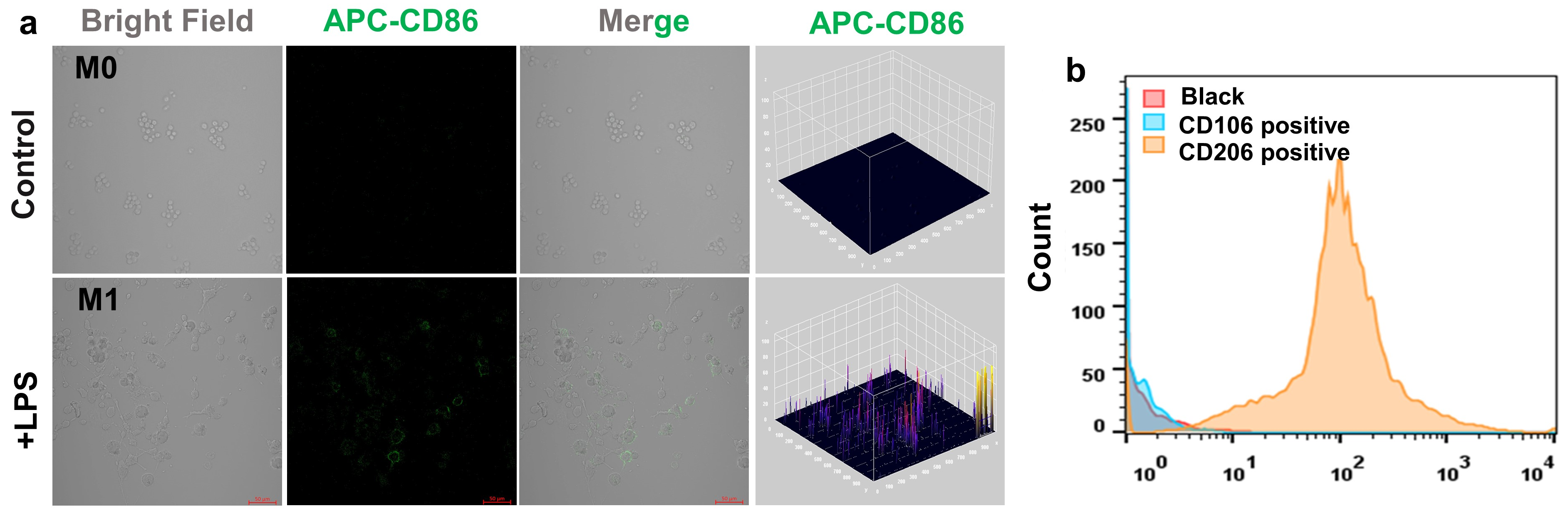


**Figure S2.** **CLSM images and flow cytometric analysis of M1/M2 macrophage markers.** a) CLSM images of M1 (APC-anti-mouse CD86) macrophage polarization. b) Flow cytometric analysis of the M2 macrophage markers CD106 and CD206.

**
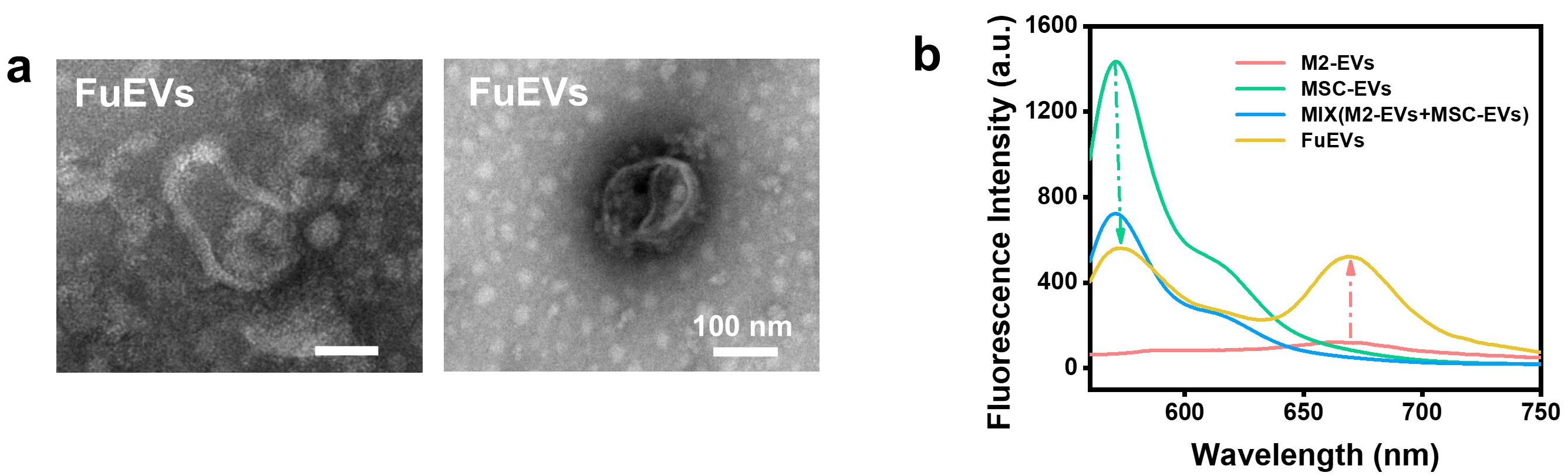
**

**Figure S3.** **TEM morphological images and fluorescence resonance energy transfer (FRET)** **results of EVs.** a) TEM morphological characterization of fusion extracellular vesicles (FuEVs). Scale bar: 100 nm. b) FRET results of three EVs (M2-EVs, MSC-EVs, and FuEVs).

**Table S1.** Drug encapsulation efficiency and drug loading efficiency of HGF and PPC.

|  | Drug Encapsulation Efficiency | Drug Loading Efficiency |
| --- | --- | --- |
| HGF | 82.5 ± 7.4% | 12.1 ± 1% |
| PPC | 61.3 ± 6.8% | 3.9 ± 0.4% |


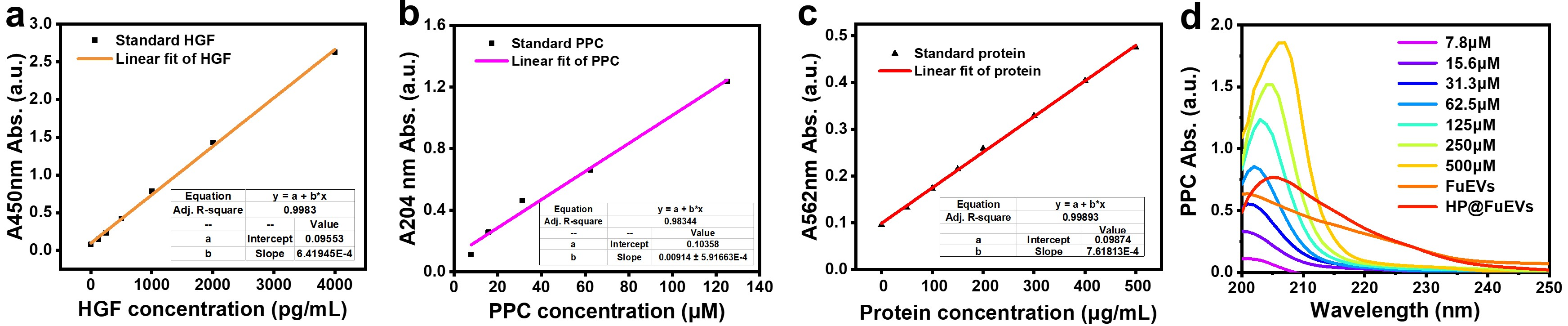


**Figure S4. The standard absorption curves for the concentrations of hepatocyte growth factor (HGF), polyunsaturated phosphatidylcholine (PPC), and standard protein.** (a) The standard absorption curve for HGF concentration. (b) The standard absorption curve for PPC. (c) The standard absorption curve for standard protein. (d) The UV absorption curves of PPC at different concentrations.


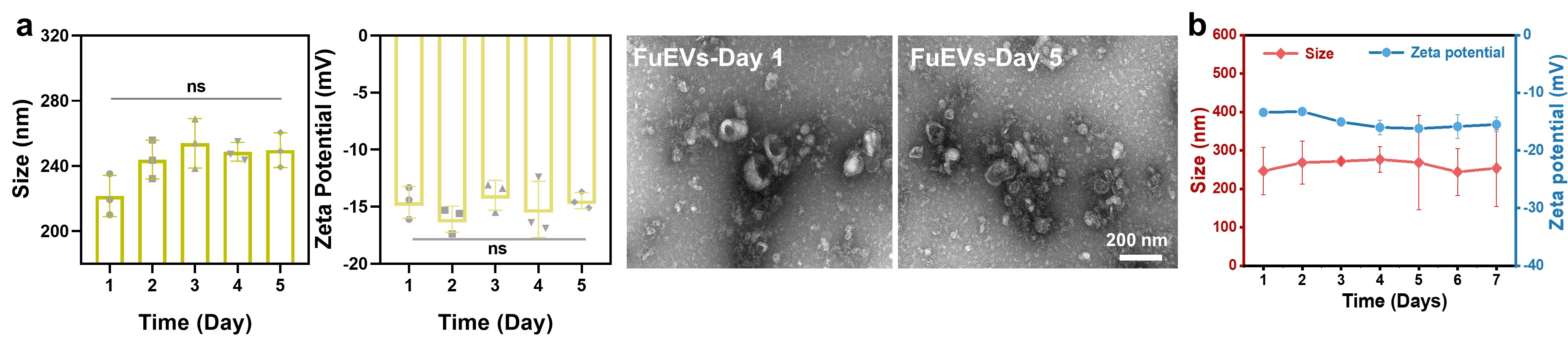


**Figure S5.** Stability of a) FuEVs (particle size; zeta potential; TEM morphology characterization) and b) FAND^HP@FuEVs^ (size and zeta potential).


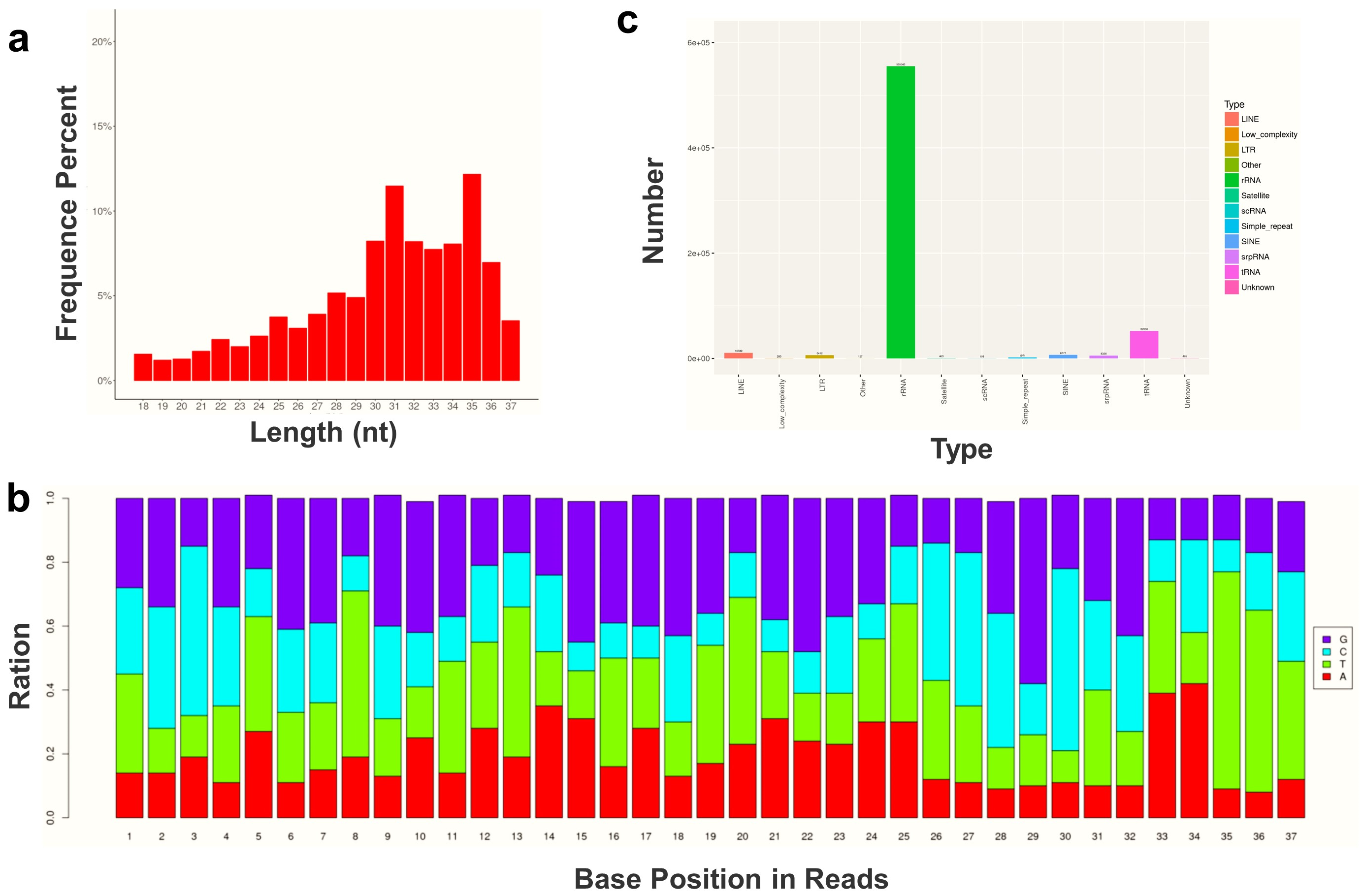


**Figure S6. Quality analysis of sequencing data for fusion vesicles (FuEVs) and M2-EVs.** (a) Sequence length distribution. (b) Base usage preference of microRNAs. (c) Classification statistics of repetitive sequences.


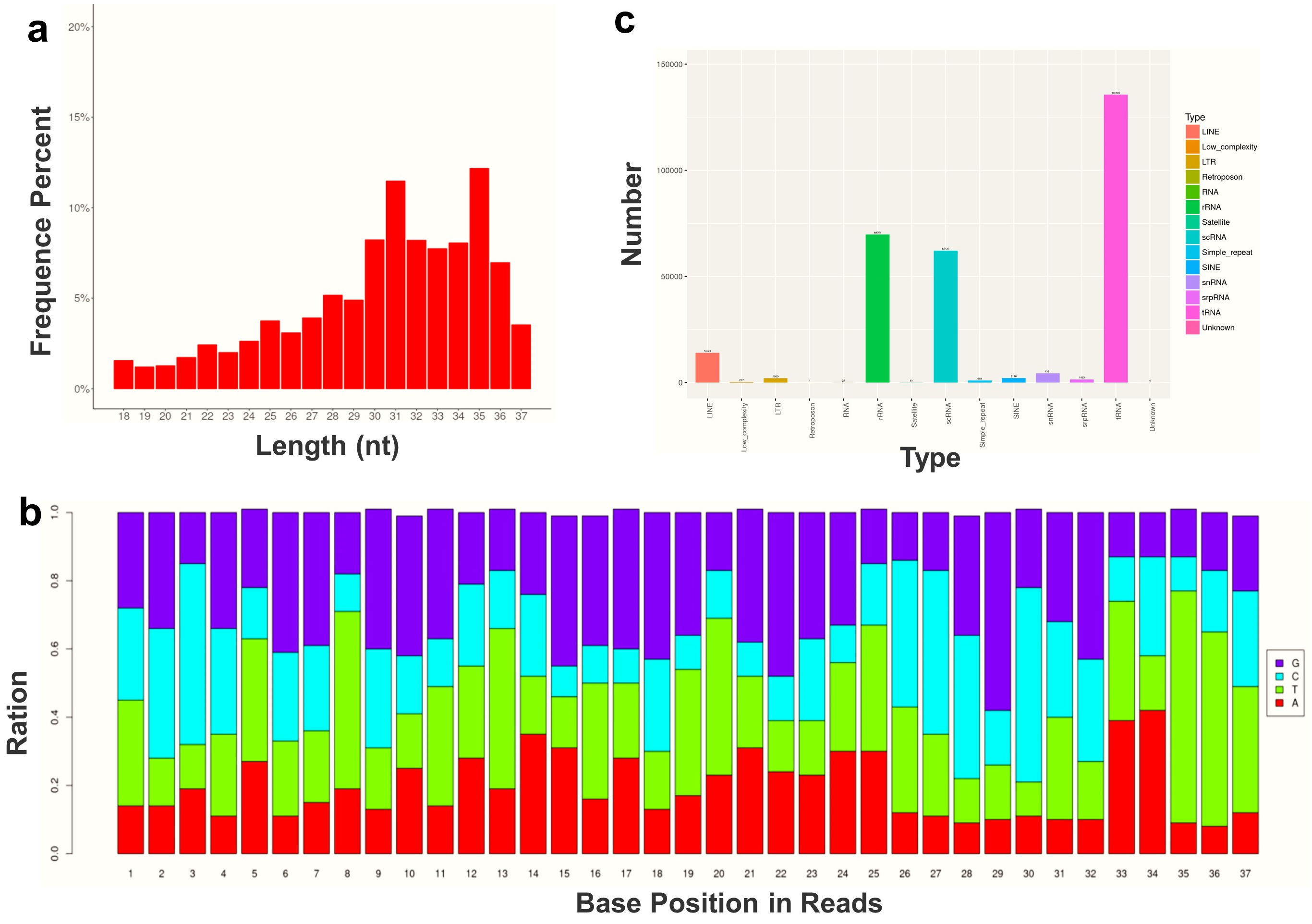


**Figure S7. Quality analysis of sequencing data for fusion vesicles (FuEVs) and MSC-EVs.** (a) Sequence length distribution. (b) Base usage preference of microRNAs. (c) Classification statistics of repetitive sequences.

**
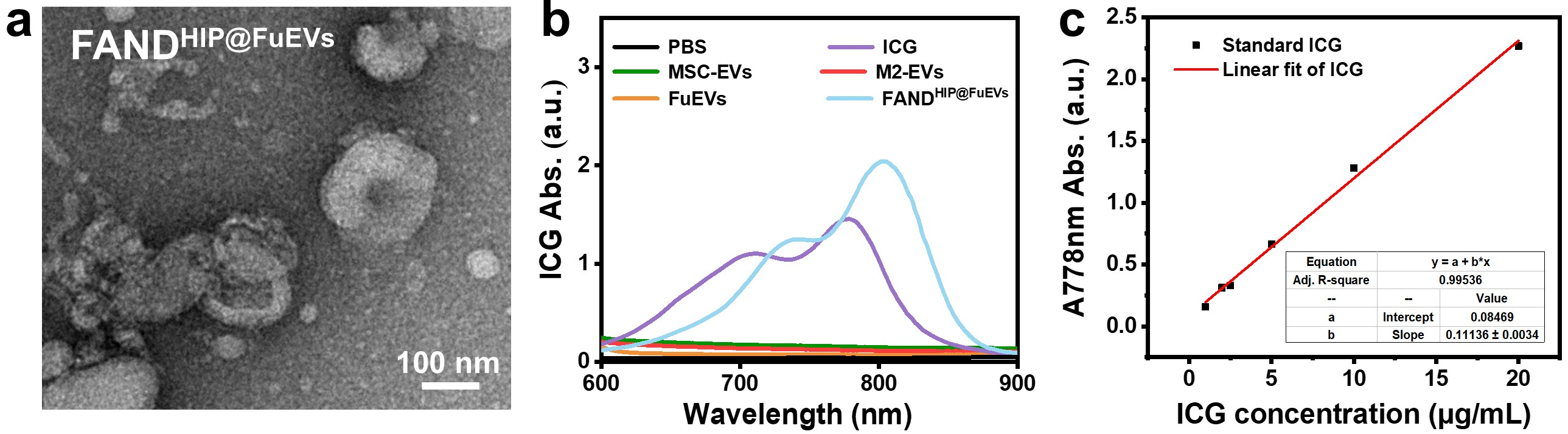
**

**Figure S8. Characterization and drug loading identification of ICG-loaded FAND^HIP@FuEVs^.** (a) TEM morphological characterization of FAND^HIP@FuEVs^. (b) UV absorption curves of FAND^HIP@FuEVs^ and each component. (c) The standard absorption curve for indocyanine green (ICG) concentration.


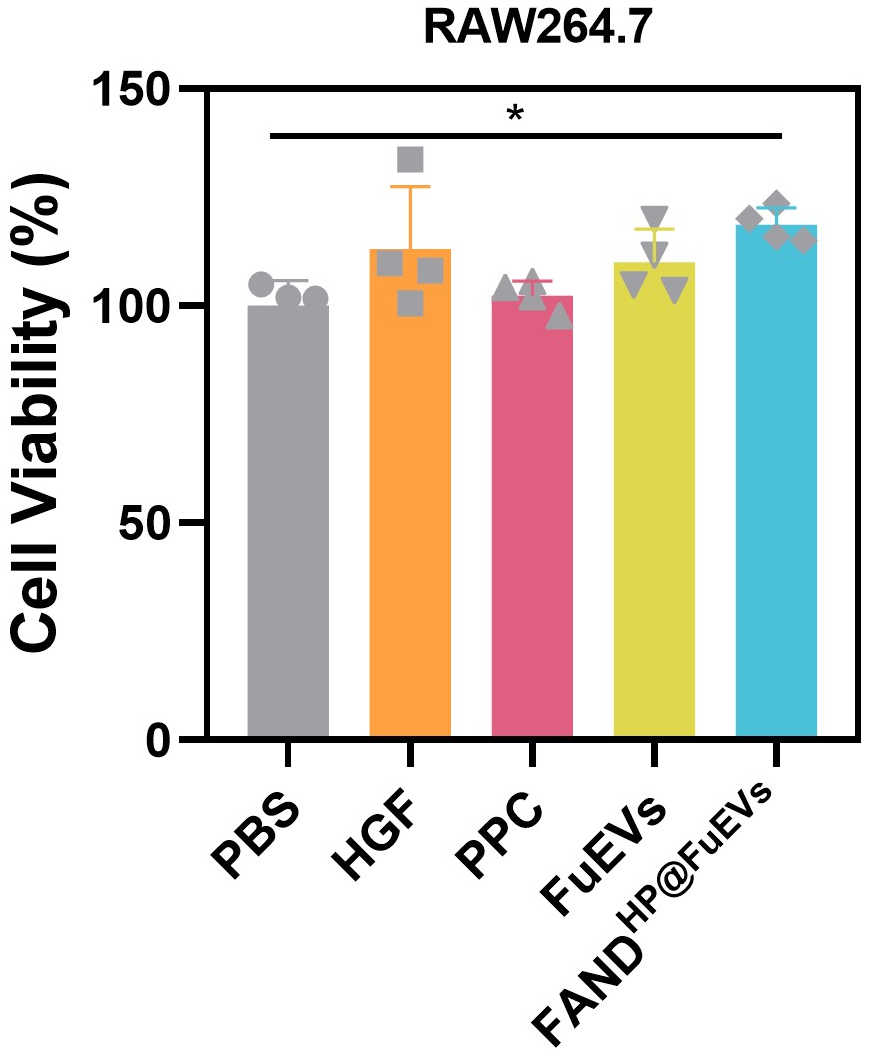


**Figure S9.** Cell viability of RAW264.7 under different treatment components (PBS, HGF, PPC, FuEVs, FAND^HP@FuEVs^) induction.


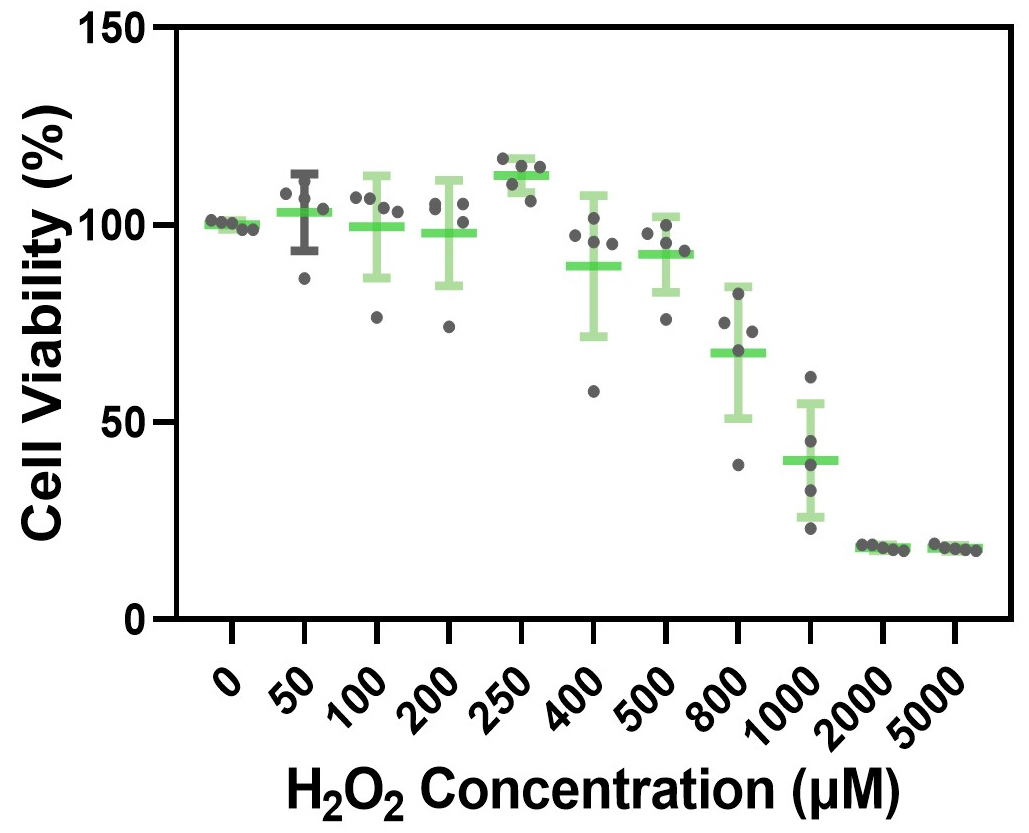


**Figure S10.** Cell viability of AML12 under different concentrations (0, 50, 100, 200, 250, 400, 500, 800, 1000, 2000, 5000 μM) of hydrogen peroxide (H_2_O_2_) induction.


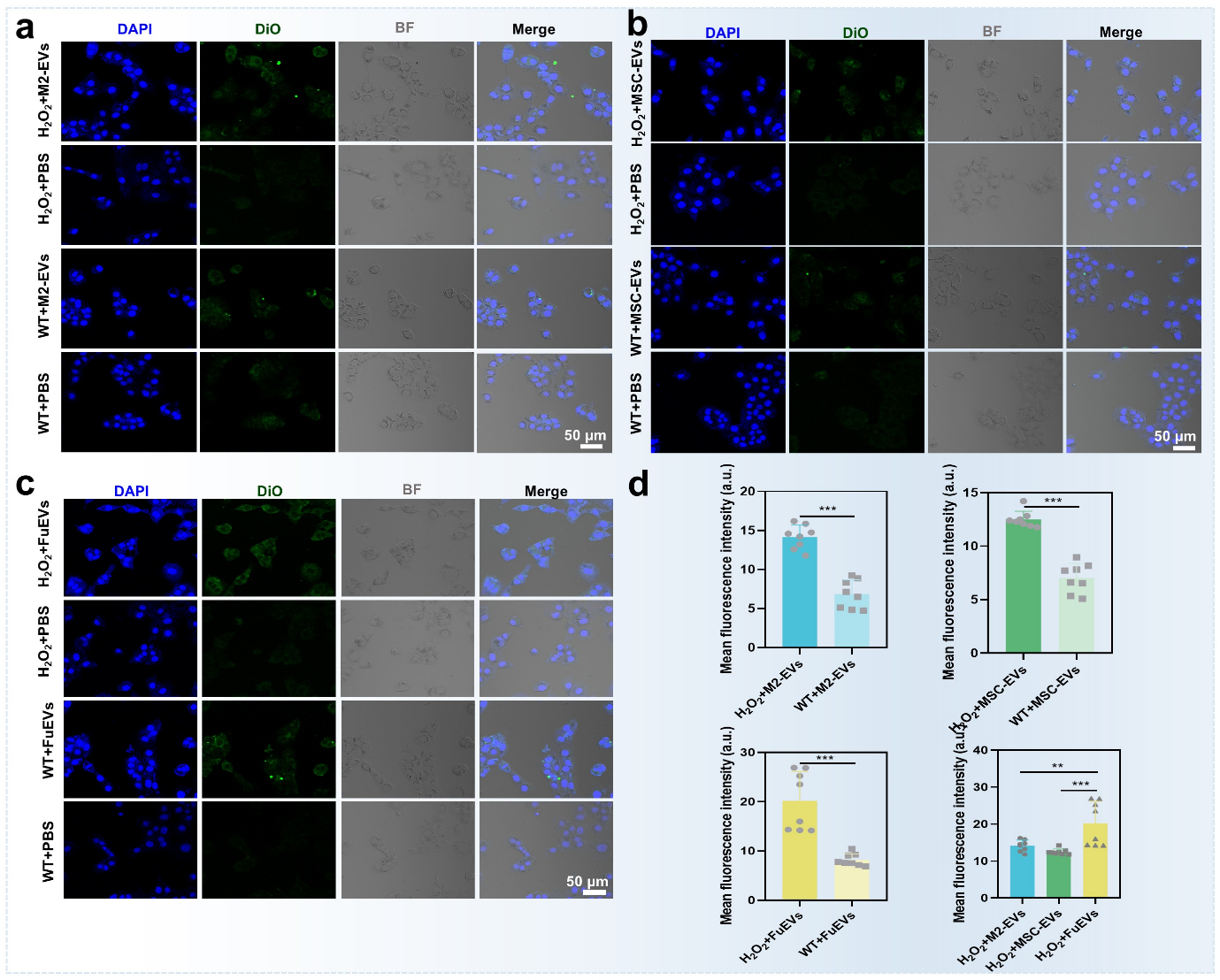


**Figure S11.** Confocal microscopy images of (a) M2-EVs, (b) MSC-EVs, and (c) FuEVs internalization in normal and inflammatory AML12 hepatocytes (scale bars: 50 μm). (d) Quantification of DiO-labeled EVs fluorescence intensity. *P < 0.05; **P < 0.01; ***P < 0.001.

**
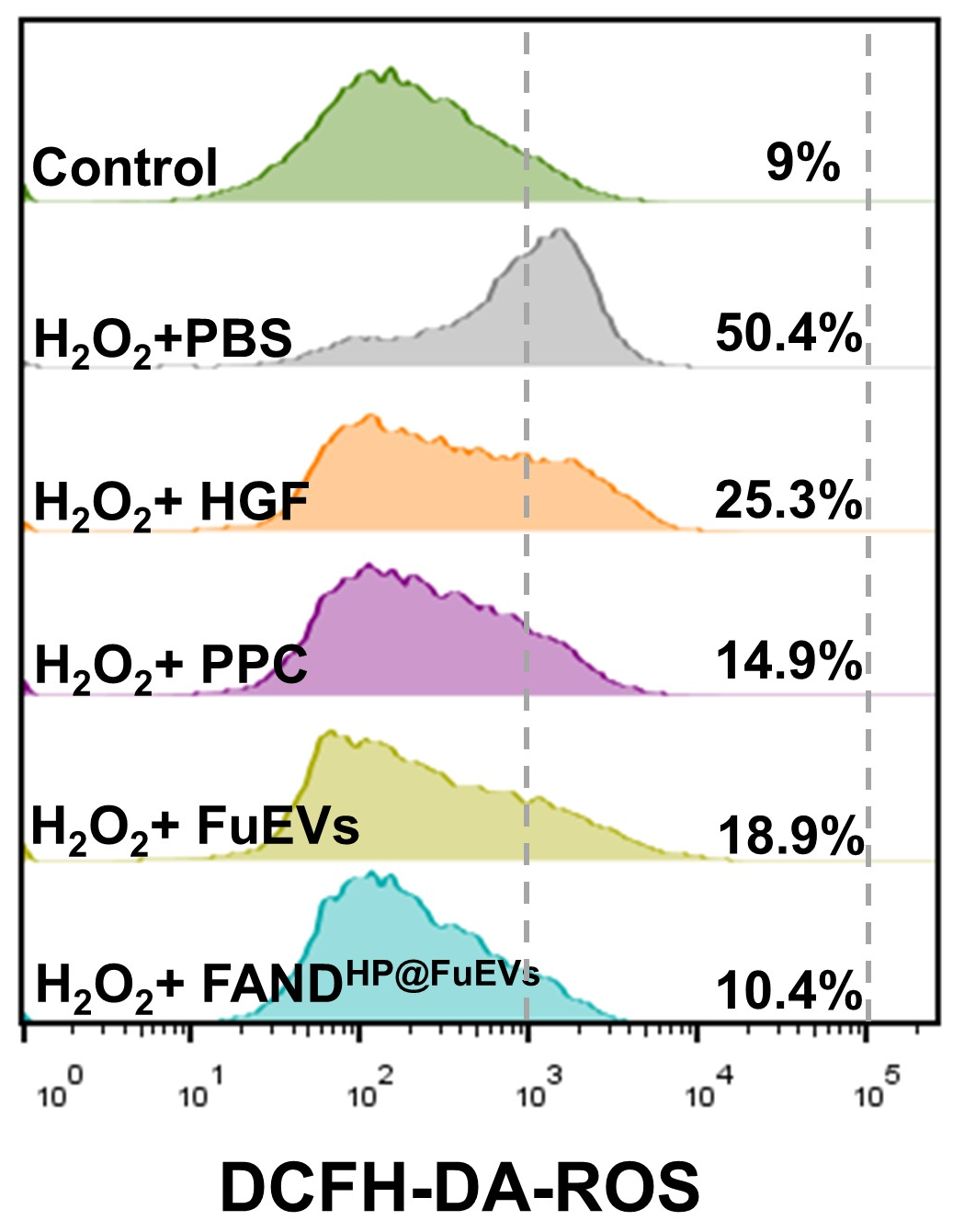
**

**Figure S12.** Flow cytometric analysis of ROS scavenging effects in each treatment group.


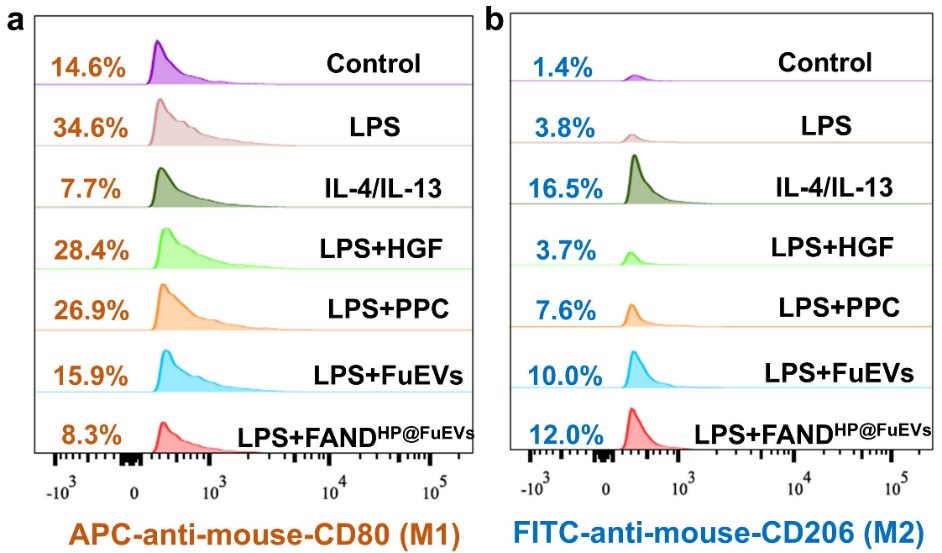


**Figure S13. Flow cytometric analysis of the macrophage repolarization effects in each treatment group.** (a) Flow cytometric analysis of the changes in CD80 levels in each treatment group. (b) Flow cytometric analysis of the changes in CD206 levels in each treatment group.


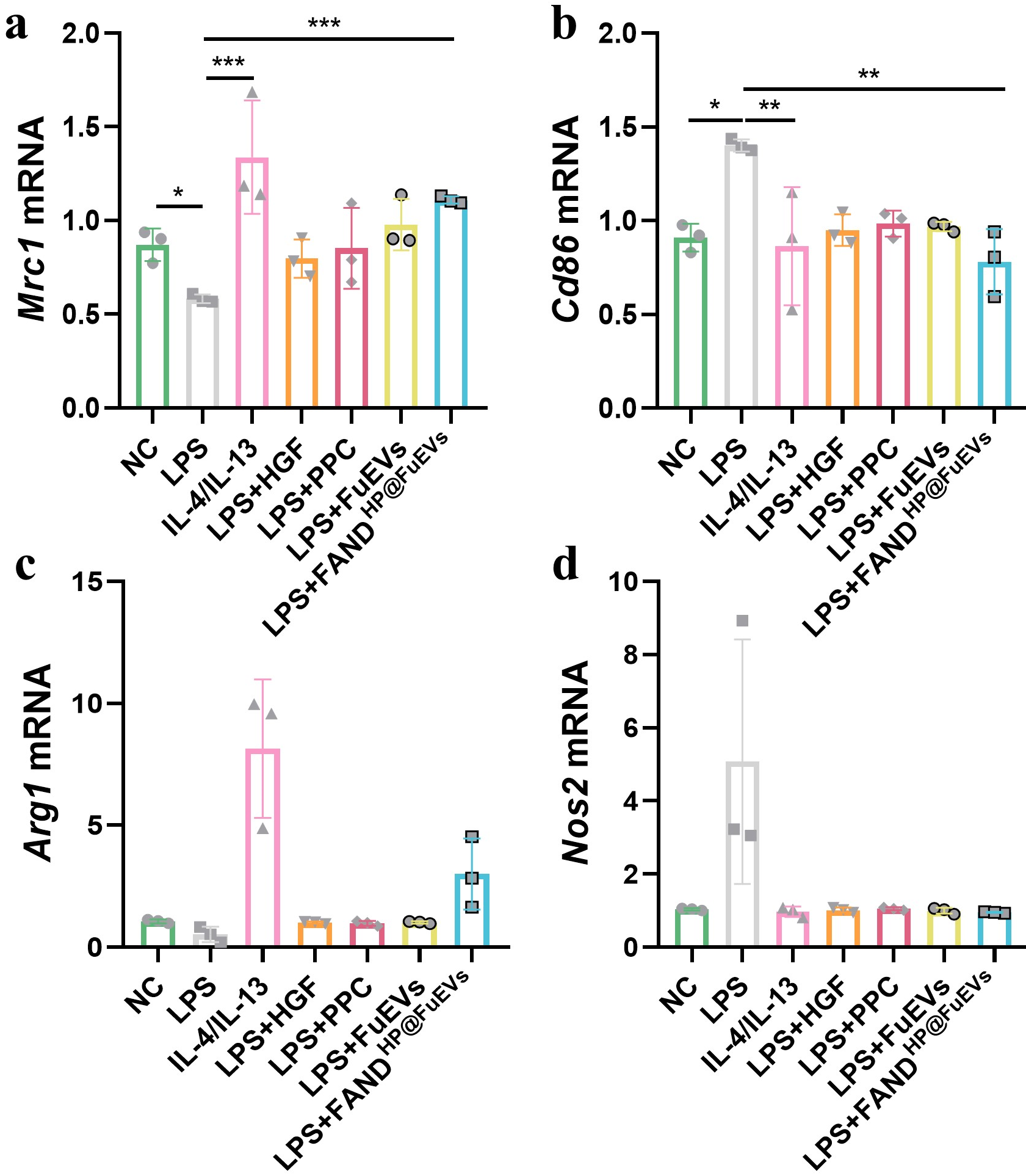


**Figure S14.** qRT-PCR results of the marker genes ((a) *Mrc1*: CD206; (b) *Cd86*: CD86; (c)*Arg1*: Arginase-1; (d) *Nos2*: inducible nitric oxide synthase) of M1/M2 from different groups (n = 3/group) *P < 0.05; **P < 0.01; ***P < 0.001.


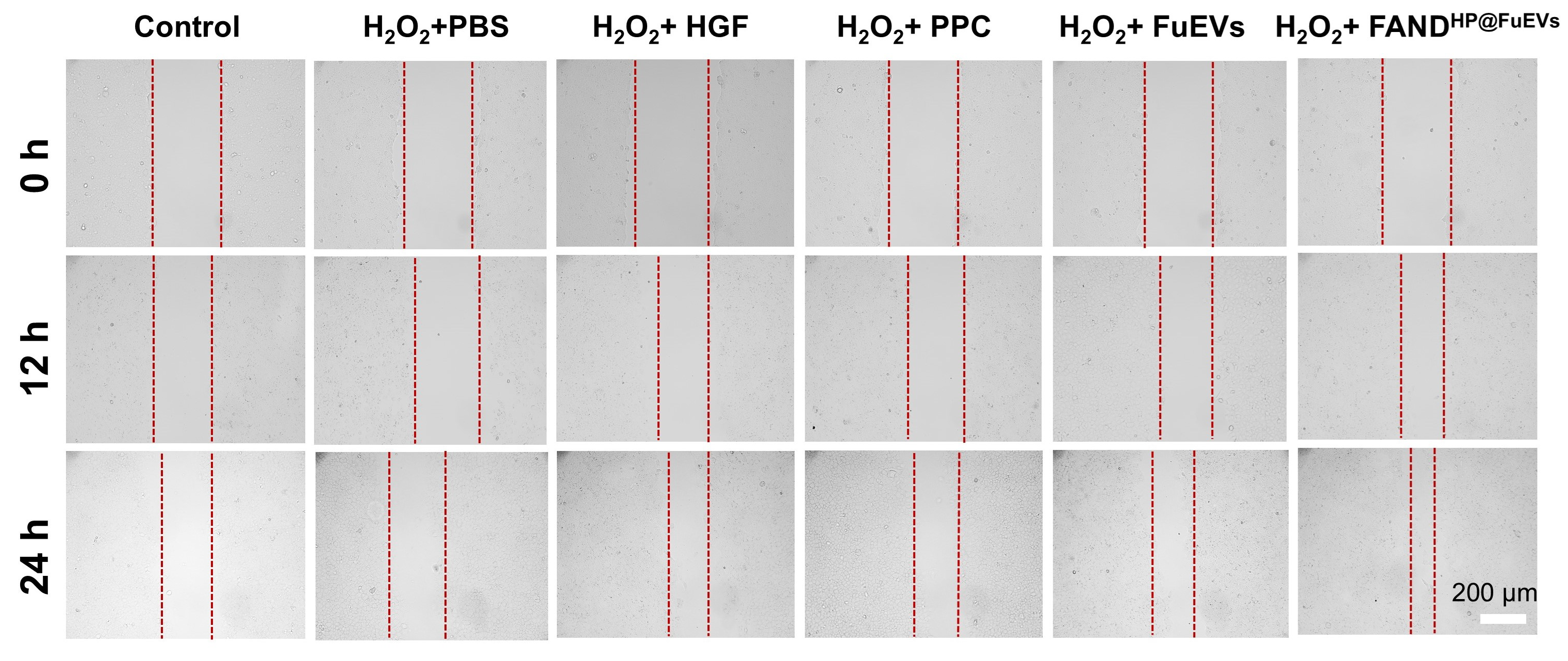


**Figure S15.** Changes in the scratch area of inflammatory AML12 cells treated with FAND^HP@FuEVs^ and various components.


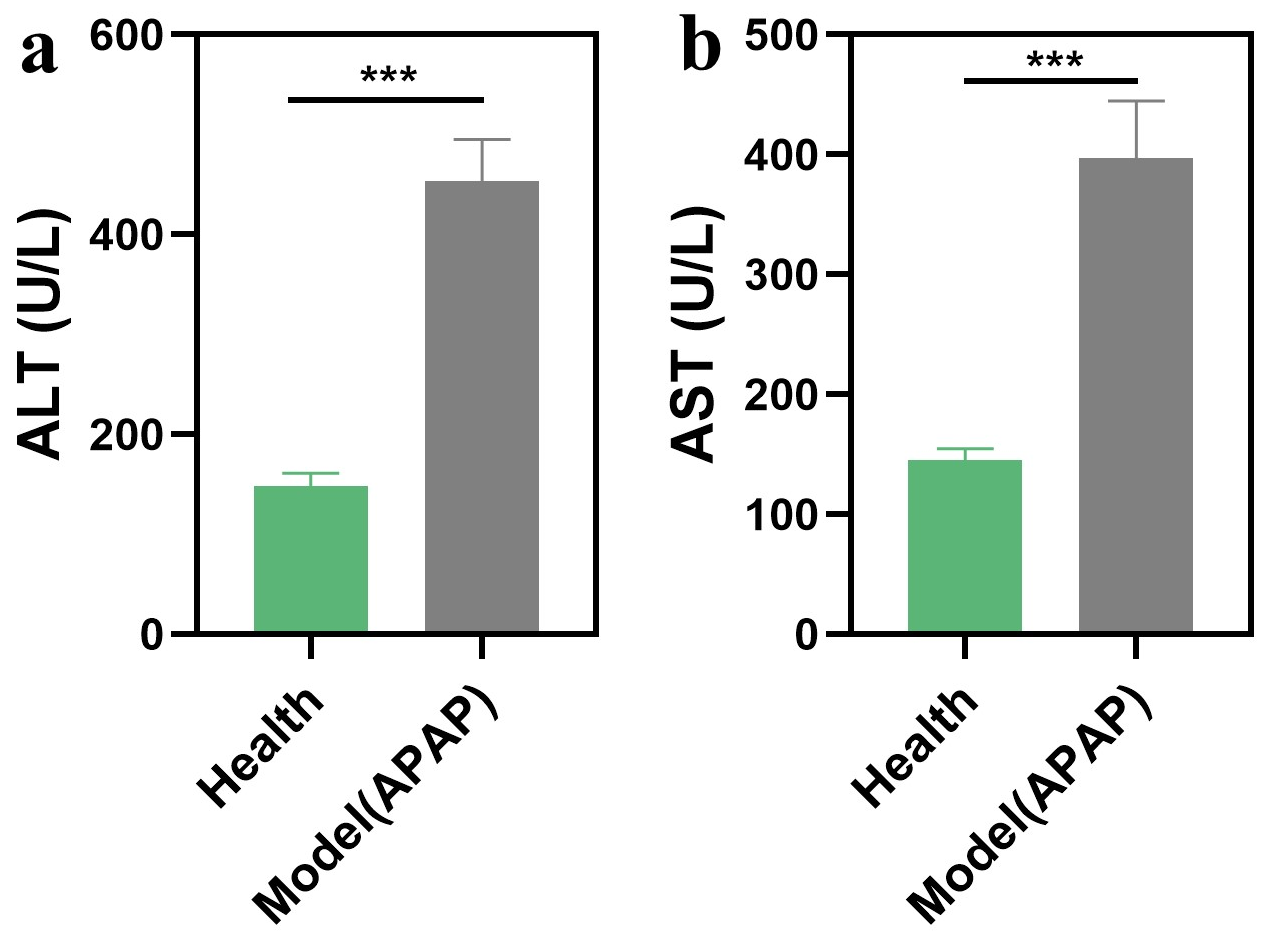


**Figure S16.** **Serum biochemical validation of acetaminophen-induced acute liver failure (ALF) model.** (a) Comparison of alanine aminotransferase (ALT) content between the healthy group and model group. (b) Comparison of aspartate aminotransferase (AST) content between the healthy group and model group. *P < 0.05; **P < 0.01; ***P < 0.001.


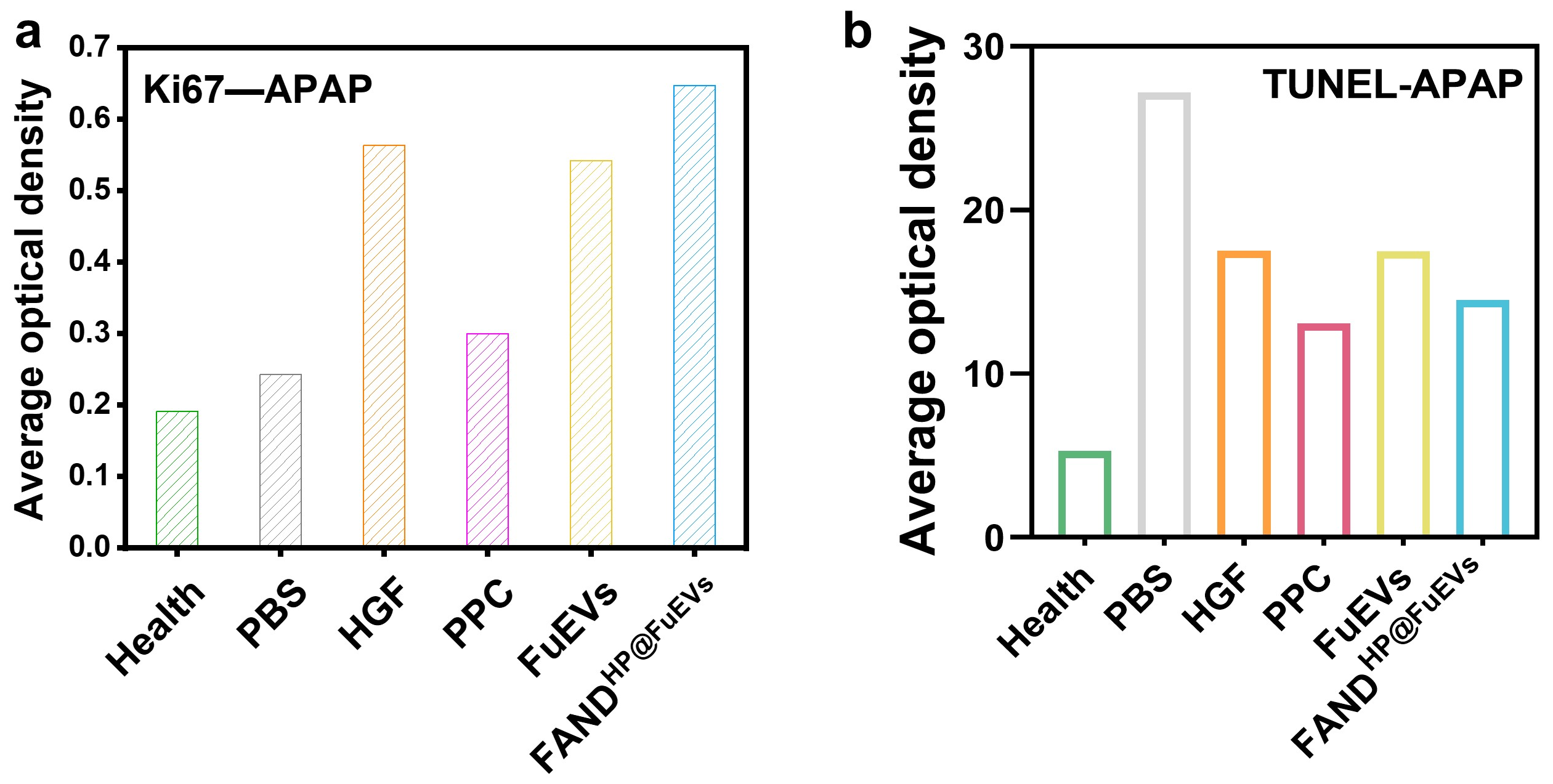


**Figure S17.** Average optical density of (a) cell proliferation (Ki67 staining) and (b) cell apoptosis (TUNEL staining) in liver tissue from acute liver failure (ALF) induced by acetaminophen (APAP).


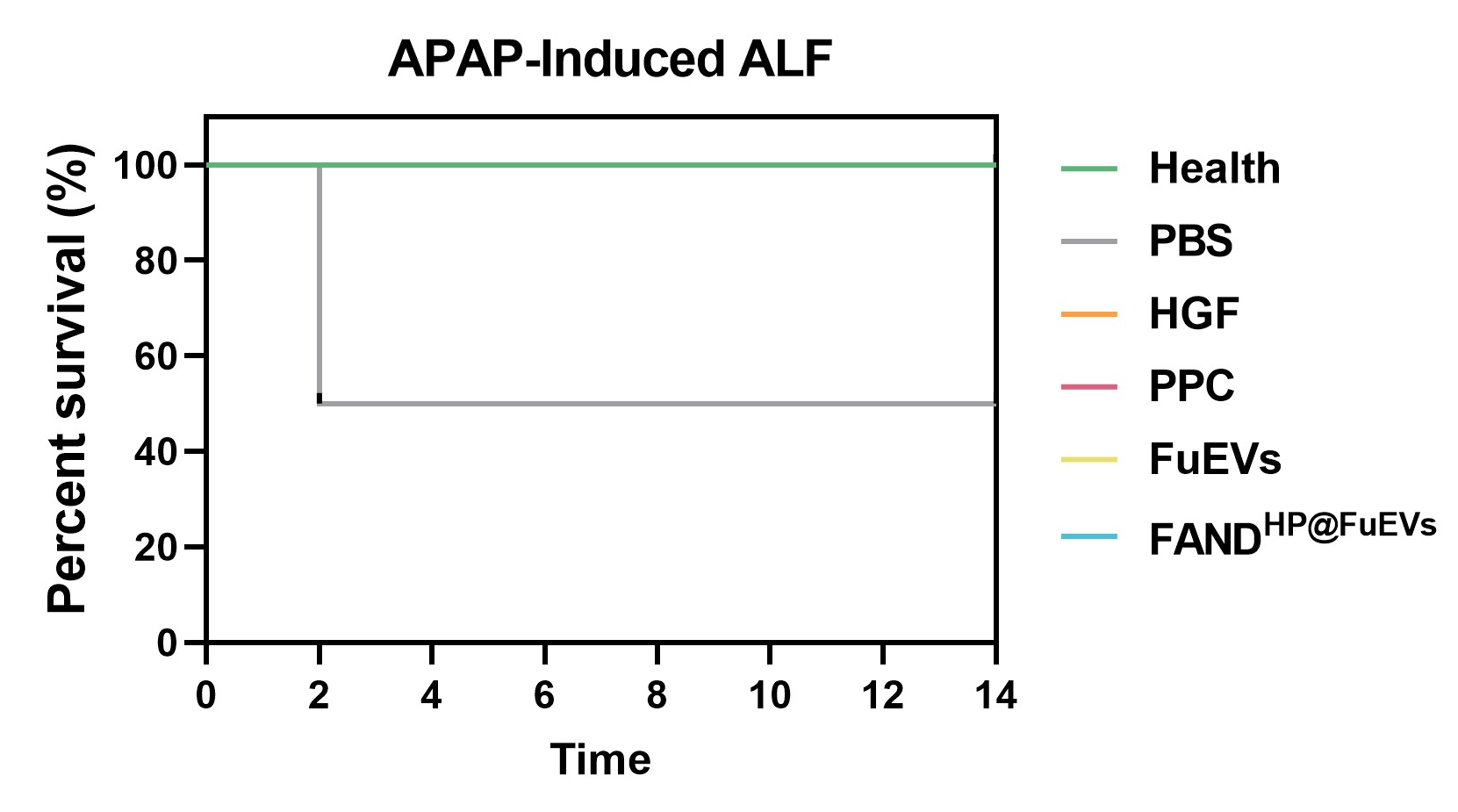


**Figure S18.** Survival curves of APAP-induced ALF mice over 14 days.


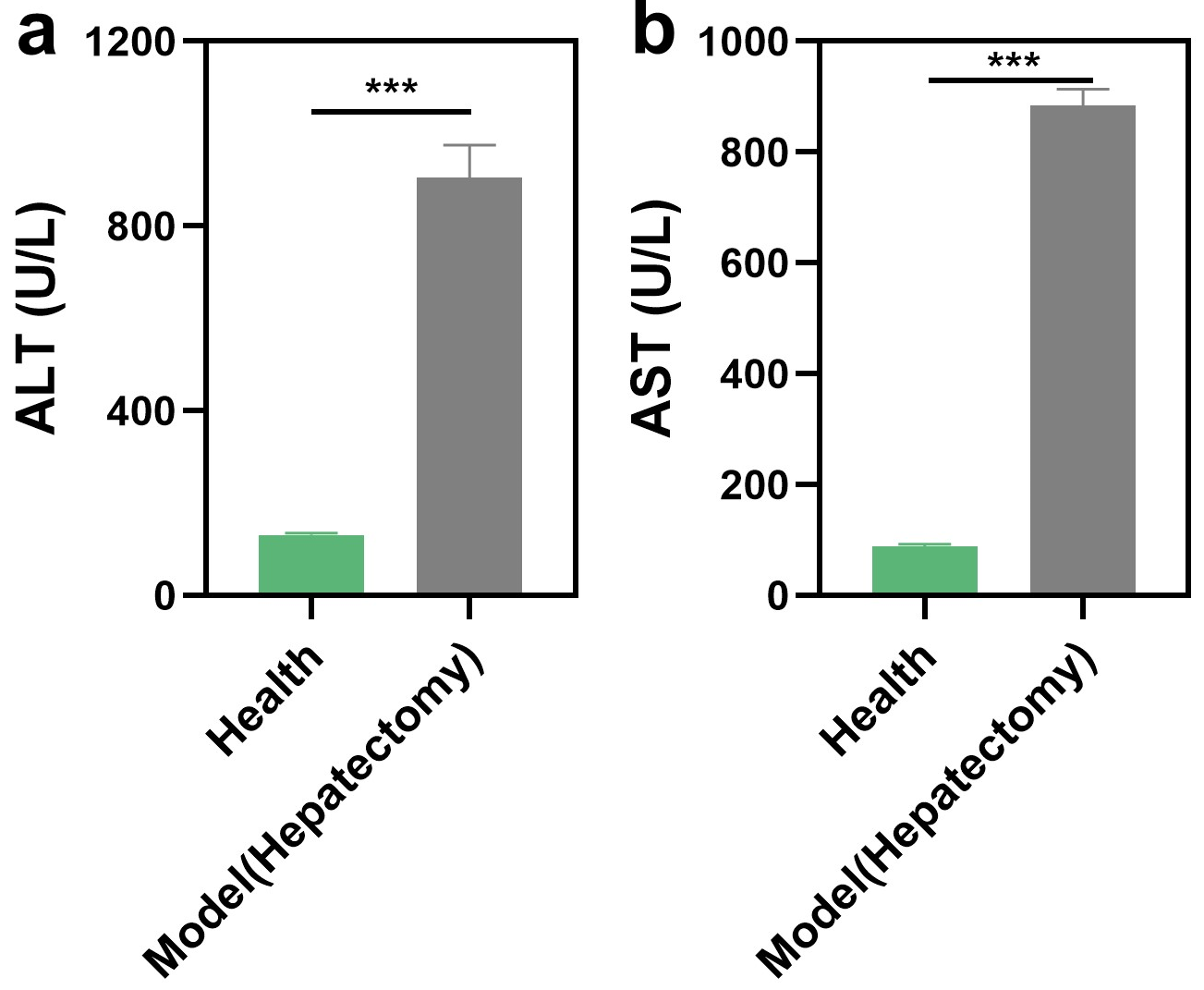


**Figure S19. Serum biochemical validation of hepatectomy-induced acute liver failure (ALF) model.** (a) Comparison of alanine aminotransferase (ALT) content between the healthy group and model group. (b) Comparison of aspartate aminotransferase (AST) content between the healthy group and model group. *P < 0.05; **P < 0.01; ***P < 0.001.


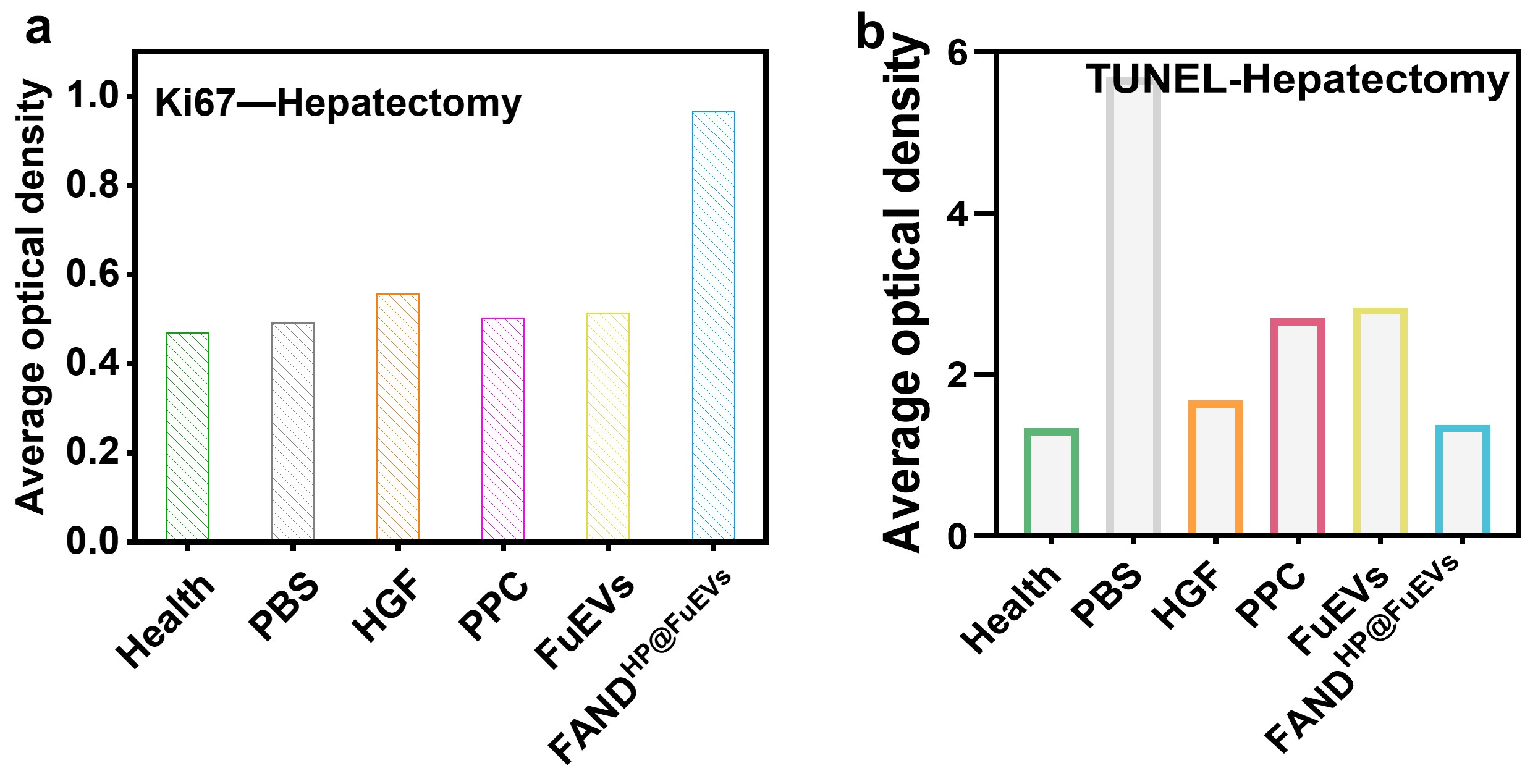


**Figure S20.** Average optical density of (a) cell proliferation (Ki67 staining) and (b) cell apoptosis (TUNEL staining) in liver tissue from acute liver failure (ALF) induced by hepatectomy.


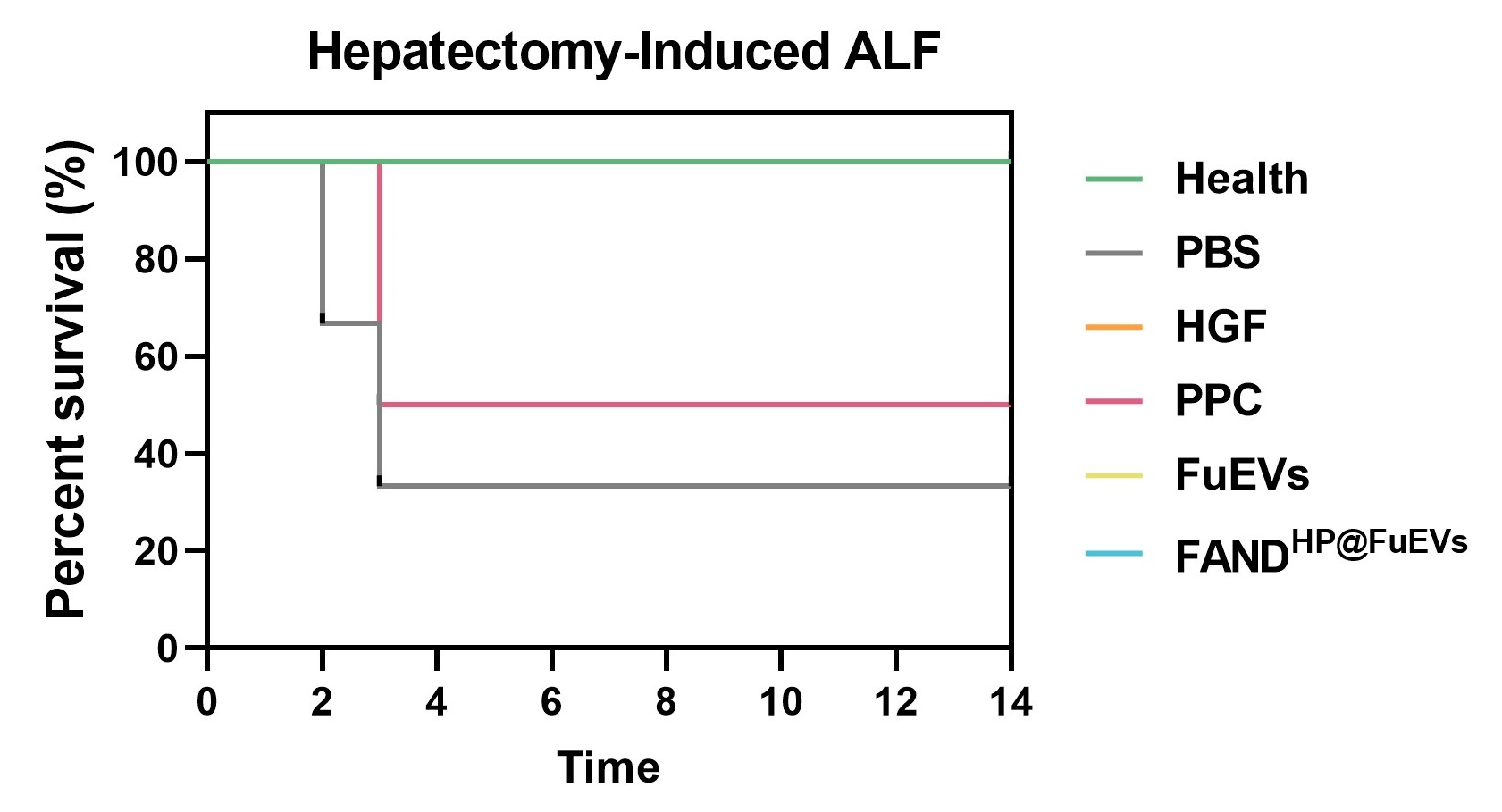


**Figure S21.** Survival curves of hepatectomy-induced ALF mice over 14 days.


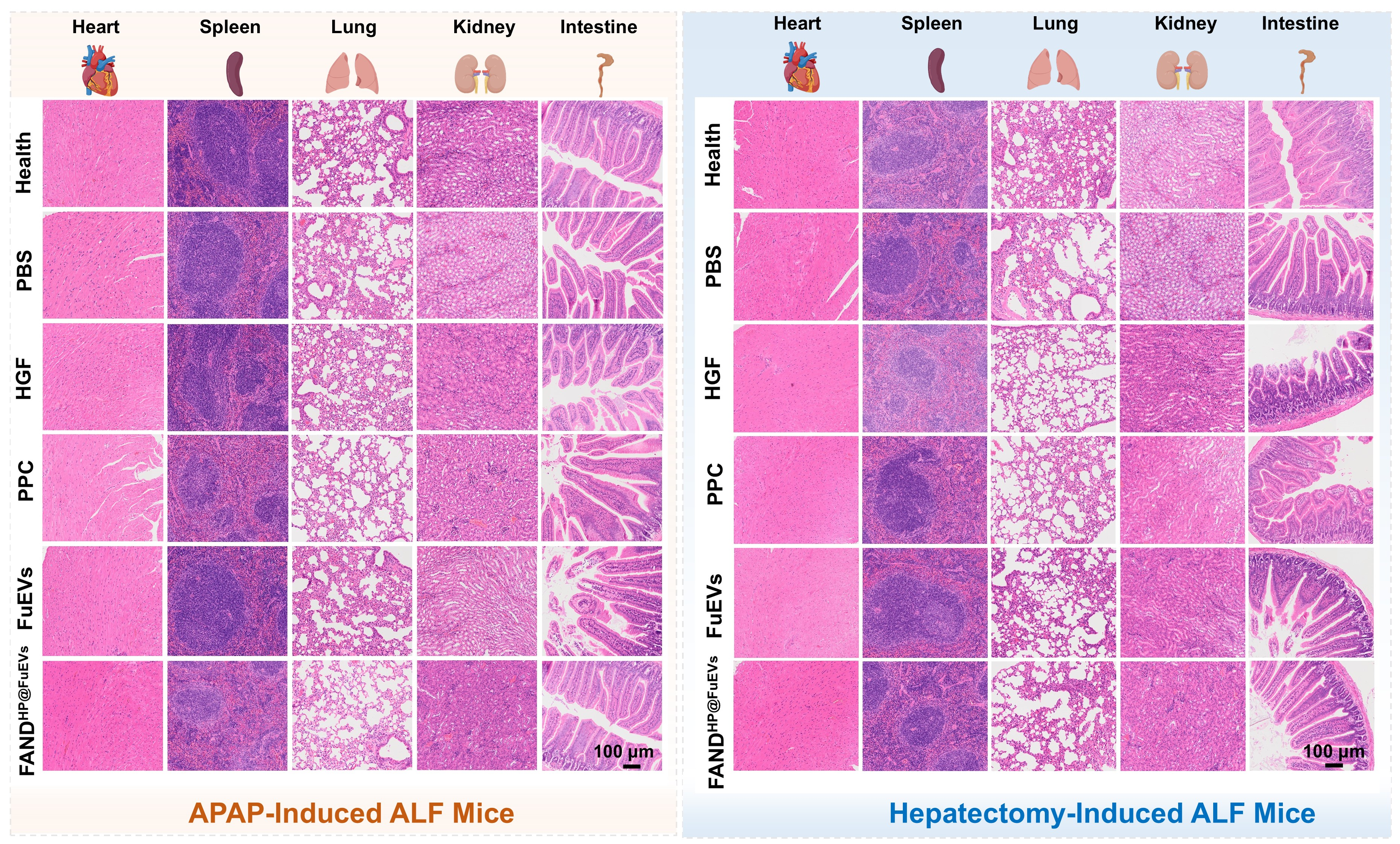


**Figure S22.** H&E-stained sections of heart, spleen, lung, and kidney from a) APAP-induced and b) hepatectomy-induced ALF mice after treatment (Health, PBS, HGF, PPC, FuEVs, and FAND^HP@FuEVs^).


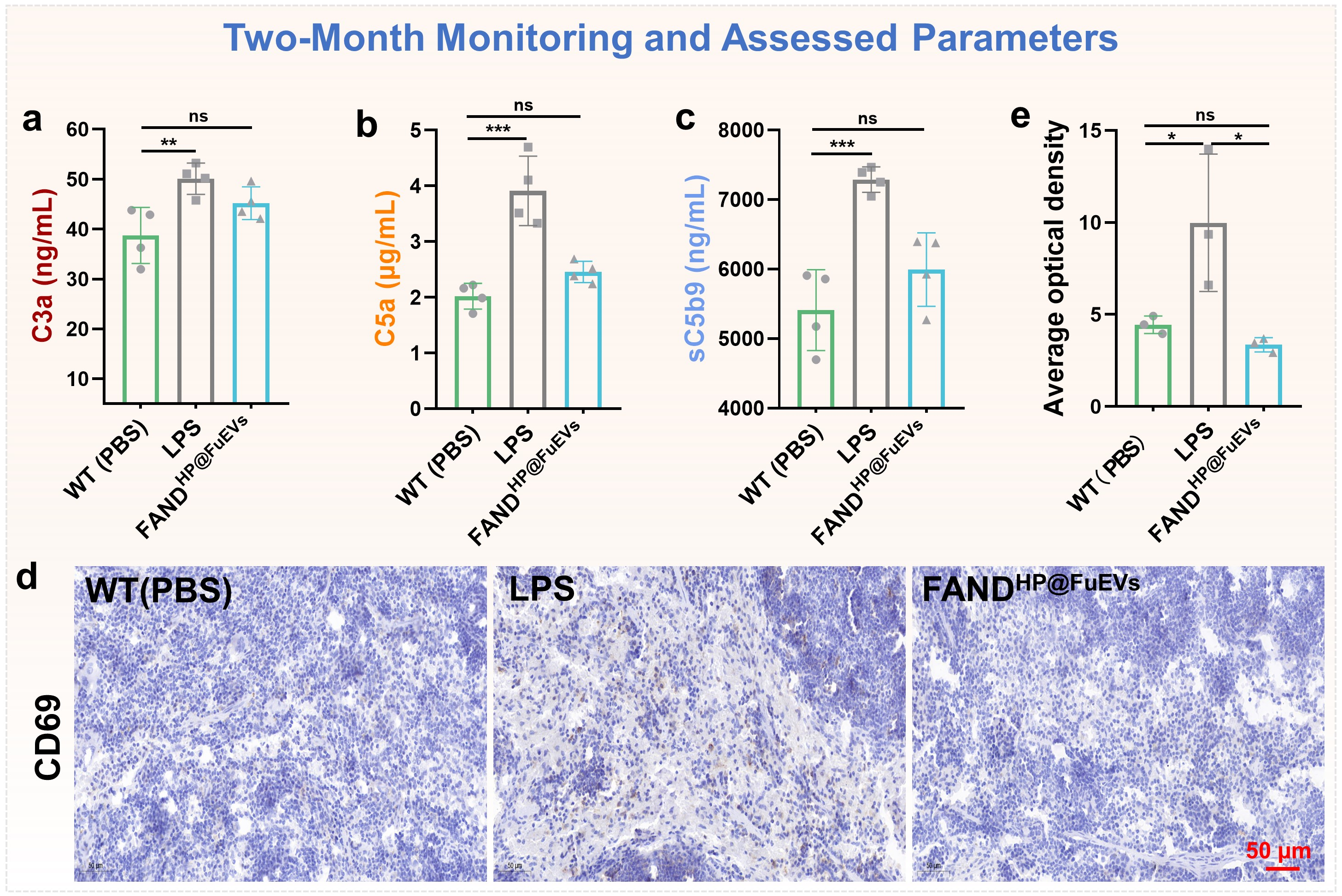


**Figure S23.** Immunogenicity assessment of FAND^HP@FuEVs^. Comparison of complement activation markers: a) C3a, b) C5a, and c) sC5b-9 among healthy mice (PBS group), LPS-injected group, and FAND^HP@FuEVs^ injection group. d) Immunohistochemical sections (CD69 staining) from healthy mice (PBS group), LPS-injected group, and FAND^HP@FuEVs^ injection group. e) Average optical density analysis of CD69 staining intensity. *P < 0.05; **P < 0.01; ***P < 0.001.
